# DIG-MambaNet: A Dual-Path Interactive Guided Mamba Network for Medical Image Segmentation

**DOI:** 10.3390/jimaging12080343

**Published:** 2026-07-28

**Authors:** Yongkang Zhu, Tianyue Yu, Hongmei Li, Xin Shu

**Affiliations:** School of Computer Science, Jiangsu University of Science and Technology, Zhenjiang 212100, China; 232241807236@stu.just.edu.cn (Y.Z.); 232241807230@stu.just.edu.cn (T.Y.)

**Keywords:** medical image segmentation, Mamba, dual-path complementary modeling block, source image-guided module, inter-layer detail refinement fusion module

## Abstract

Reliable medical image segmentation remains challenging because models must preserve fine boundary details while maintaining global semantic consistency. CNNs capture local structures effectively but have limited long-range modeling ability, whereas Transformer-based methods improve global context at high computational cost. Mamba-based state space models offer efficient long-range modeling, but may weaken high-frequency textures and boundary cues. To address these limitations, we propose DIG-MambaNet, a Dual-path Interactive Guided Mamba Network for medical image segmentation. The network introduces a dual-path complementary modeling block (DCM Block), where a cross-feature spatial interaction module (CSIM) adaptively integrates CNN-based local features and Mamba-based global features. A source image-guided module (SIGM) injects high-frequency information from the original image to compensate for downsampling-induced detail loss, while an inter-layer detail refinement fusion module (IDRFM) improves encoder–decoder feature alignment during reconstruction. Experiments on 2018DSB, ISIC2018, JSUAH-Cerebellum, and CVC-ClinicDB, covering nuclei segmentation in microscopy images, skin lesion segmentation in dermoscopic images, fetal cerebellum segmentation in ultrasound images, and polyp segmentation in colonoscopy images, demonstrate that DIG-MambaNet achieves consistent and competitive performance across diverse target structures and imaging conditions, with improved boundary delineation and favorable overlap-based accuracy compared with representative CNN-, Transformer-, and Mamba-based methods.

## 1. Introduction

Segmentation is a fundamental task in medical image analysis and aims to delineate clinically relevant anatomical structures and lesions, including organ boundaries, tumors, and abnormal tissues. Accurate segmentation supports quantitative diagnosis, preoperative planning, treatment evaluation, and follow-up assessment. In clinical practice, however, medical images are often affected by noise, artifacts, low contrast, blurred boundaries, and large variations in target scale. Images acquired from different devices or centers may also exhibit distribution shifts, further reducing segmentation reliability. These challenges require models to preserve fine local structures while maintaining global semantic consistency. Therefore, effective integration of local detail representation and global contextual modeling remains a central problem in medical image segmentation [1].

Convolutional neural networks (CNNs) have been widely used for medical image segmentation because of their strong local feature extraction capability. The classical U-Net [2] adopts an encoder–decoder architecture with skip connections to preserve spatial details and has served as the foundation for many subsequent variants. U-Net++ [3] introduces nested dense skip connections to reduce semantic gaps between different layers, and Attention U-Net [4] uses attention gates to enhance responses in target regions. However, Luo et al. [5] showed that the effective receptive field of CNNs is often smaller than its theoretical size, limiting their ability to capture long-range dependencies. Consequently, CNN-based methods may struggle to maintain global semantic consistency, particularly in images with complex anatomical structures or low-contrast boundaries. Recent studies have introduced attention mechanisms, adaptive feature modeling, and feature refinement strategies into CNN-based frameworks. CASA-Net [6] improves feature discriminability through context-aware self-attention, AA-Net [7] enhances multi-scale representation through adaptive encoding and attention-based decoding, and HAFRN-CDP [8] reduces semantic gaps and detail loss through cross-resolution feature refinement and hierarchical fusion. Although these methods improve local representation and feature fusion, they do not fully resolve the inherent limitation of CNNs in global dependency modeling.

To overcome this limitation, Transformer-based methods introduce self-attention into visual representation learning. The Vision Transformer (ViT) [9] models global semantic relationships through interactions among image patches, and several Transformer-based frameworks have been developed for medical image segmentation [10]. TransUNet [11] combines the global encoding capability of Transformers with the local feature extraction ability of CNNs. Swin-Unet [12] adopts shifted window attention to construct a hierarchical U-shaped network for multi-scale feature modeling. MedT [13] uses gated axial attention to alleviate the difficulty of limited pretraining data in medical imaging. Despite their effectiveness in global context modeling, Transformer-based methods require substantial computational and memory resources, especially for high-resolution clinical images, which restricts their practical use in resource-constrained settings.

State space models (SSMs), particularly Mamba [14], have recently attracted increasing attention as an efficient alternative for long-range dependency modeling. By introducing a hardware-aware selective scanning mechanism, Mamba can model long-range dependencies with near-linear computational complexity, making it promising for high-resolution visual tasks. However, Mamba-based architectures still have limitations in local structure representation. Unlike convolutional operations, Mamba does not inherently emphasize high-frequency textures or fine-grained boundary cues, which may reduce its stability in identifying small lesions and delineating complex anatomical structures. Recent studies have therefore explored hybrid CNN-Mamba designs to combine CNN-based local detail extraction with Mamba-based global semantic modeling. U-Mamba [15] adopts a serial combination of convolutional modules and Mamba sequences, whereas MedMamba [16] and HC-Mamba [17] construct hybrid feature extraction units with separate CNN and SSM branches. Although these methods improve segmentation performance, local-global feature interaction remains relatively coarse. Most rely on static fusion strategies, such as channel concatenation or element-wise addition, which are insufficient for dynamically modeling the complementary relationship between local and global features. In addition, successive downsampling in deep hierarchical networks gradually weakens multi-scale spatial structures and high-frequency boundary information from the original image. Existing hybrid models usually lack explicit mechanisms to compensate for this detail loss using source image information. During feature reconstruction, simple skip connections may also fail to bridge semantic gaps between the encoder and decoder, leading to blurred boundaries or fragmented regions in complex segmentation tasks.

To address these challenges, we propose DIG-MambaNet, a Dual-path Interactive Guided Mamba Network for medical image segmentation. The network is designed to jointly model local details and global semantics through multi-scale feature interaction and explicit spatial guidance. Specifically, we develop a dual-path complementary modeling block (DCM Block), in which a cross-feature spatial interaction module (CSIM) enables adaptive interaction between local CNN features and global Mamba features. Through spatially adaptive weighting, CSIM integrates CNN-based texture cues with Mamba-based contextual representations. To compensate for the loss of spatial details caused by successive downsampling, we introduce a source image-guided module (SIGM), which extracts high-frequency information from the original input and injects it into deep feature maps through a gating mechanism. To further reduce semantic gaps between the encoder and decoder, an inter-layer detail refinement fusion module (IDRFM) is embedded in the skip connections. IDRFM uses high-resolution encoder features to guide the refinement of low-resolution semantic features, improving spatial alignment and semantic consistency during feature recovery. Overall, DIG-MambaNet coordinates local texture representation, global context modeling, and cross-layer detail refinement, providing a targeted framework for complex medical image segmentation.

The main contributions of this work are summarized as follows:

- We propose DIG-MambaNet, a medical image segmentation network that integrates CNN and Mamba in a dual-path architecture. A DCM Block with CSIM is developed to adaptively fuse local and global features, improving the joint representation of fine-grained structures and global semantic context.
- We introduce SIGM to extract high-frequency spatial information from the original input image and inject it into deep feature representations through a gating mechanism. This design alleviates the loss of fine spatial structures caused by successive downsampling and helps preserve boundary details and complex lesion structures.
- We design IDRFM to improve cross-layer feature fusion between the encoder and decoder. By using high-resolution encoder features to guide the refinement of low-resolution semantic features, IDRFM reduces semantic gaps across layers and improves spatial alignment during feature recovery.
- We conduct experiments on the 2018DSB, ISIC2018, JSUAH-Cerebellum, and CVC-ClinicDB datasets. The results show that DIG-MambaNet achieves competitive performance across multiple evaluation metrics, confirming its effectiveness and stability in medical image segmentation.

## 2. Related Work

### 2.1. Medical Image Segmentation

Medical image segmentation aims to delineate clinically relevant structures from complex anatomical backgrounds and has been widely studied using deep neural networks. CNN-based encoder–decoder architectures remain a major foundation in this field. Representative models, including U-Net [2], V-Net [18], and U-Net++ [3], use skip connections to combine high-resolution spatial details with deep semantic representations, which has made them effective across many segmentation tasks. Residual and dense connectivity mechanisms, such as ResNet [19] and DenseNet [20], further improve feature propagation and representation capacity for complex anatomical structures. Lightweight convolutional networks, including MobileNet [21] and MobileNetV2 [22], have also been introduced for resource-constrained scenarios. However, most CNN-based methods rely primarily on local convolutional operations, which limits their ability to capture long-range spatial dependencies and global semantic relationships.

To enhance feature discrimination within convolutional networks, attention mechanisms have been widely explored for adaptive feature recalibration. Squeeze-and-Excitation Networks introduced channel-wise attention to model inter-channel dependencies and selectively emphasize informative feature responses [23]. CBAM further extended this idea by sequentially integrating channel and spatial attention, enabling convolutional networks to refine feature representations from complementary perspectives [24]. In medical image segmentation, concurrent spatial and channel squeeze-and-excitation blocks have been incorporated into fully convolutional networks to improve the recalibration of anatomical feature maps while introducing limited additional complexity [25]. Beyond channel-spatial attention, Triplet Attention captures cross-dimensional interactions through a three-branch structure, providing a lightweight way to model dependencies among channel and spatial dimensions [26]. Coordinate Attention further embeds positional information into channel attention by separately encoding features along the horizontal and vertical directions, which improves spatially sensitive representation while maintaining efficiency [27]. These studies demonstrate that attention mechanisms can improve the selectivity and spatial awareness of CNN-based models. However, most of them still mainly act as feature recalibration modules and do not fully address the limitation of local convolution in modeling long-range dependencies and global semantic relationships in complex medical images.

To strengthen global modeling, Transformer-based architectures have been increasingly explored in medical image segmentation. UCTransNet [28] revisits skip connections in U-shaped networks and introduces a channel-wise Transformer mechanism to reduce semantic inconsistency between encoder and decoder features. Swin Transformer [29] uses shifted window attention to extract hierarchical features while partly reducing the computational burden of full self-attention. Pyramid Vision Transformer (PVT) [30] adopts a pyramid structure to improve multi-scale representation. These methods demonstrate the value of attention-based global context modeling, but self-attention still incurs substantial computational and memory costs, especially when processing high-resolution medical images. This limits the practical efficiency and scalability of Transformer-based segmentation frameworks.

### 2.2. State Space Models for Medical Image Segmentation

To reduce the computational and memory burden of self-attention on high-resolution inputs, state space models (SSMs) have recently emerged as an efficient direction for sequence modeling. Mamba [14] introduces a hardware-aware selective scanning mechanism that enables long-range dependency modeling with favorable computational efficiency. This property has motivated its extension to computer vision, including backbone networks such as Vision Mamba [31] and VMamba [32], which have shown competitive representation capability in visual recognition and dense prediction tasks.

The efficiency and long-range modeling ability of Mamba have encouraged its use in medical image segmentation. U-Mamba [15] and VM-UNet [33] integrate state space models into U-shaped segmentation frameworks to enhance global context modeling. MSVM-UNet [34] introduces multi-scale visual state space blocks with large-kernel embeddings to reduce directional sensitivity in two-dimensional image modeling and improve performance on the BraTS2023 dataset. H-vmunet [35] uses a high-order two-dimensional selective scanning mechanism (H-SS2D) to reduce redundant scan paths and improve feature modeling efficiency. UltraLight VM-UNet [36] emphasizes parameter efficiency through a parallel visual Mamba architecture, while Swin-UMamba [37] demonstrates the value of ImageNet-pretrained Mamba blocks for medical image segmentation.

Despite these advances, existing SSM-based segmentation methods still face several limitations. First, many hybrid architectures do not explicitly model the complementarity between CNN-based local details and Mamba-based global context, and their feature interaction is often limited to coarse fusion. Second, repeated downsampling can weaken source-level spatial details, including high-frequency textures and boundary cues that are critical for small lesions or ambiguous anatomical structures. Third, conventional skip connections may not sufficiently reduce semantic inconsistency between encoder and decoder features during reconstruction. These limitations can lead to blurred boundaries, fragmented regions, or reduced robustness in complex medical images with heterogeneous structures and low contrast.

Motivated by these observations, DIG-MambaNet is designed to jointly enhance local detail representation, global context modeling, and cross-layer feature alignment in an encoder–decoder segmentation framework. By combining dual-path CNN-Mamba feature modeling, source-image-guided detail compensation, and inter-layer detail refinement, the proposed method aims to address the remaining limitations of current hybrid and SSM-based segmentation models.

## 3. Method

### 3.1. Overall Architecture

**Figure 1:**
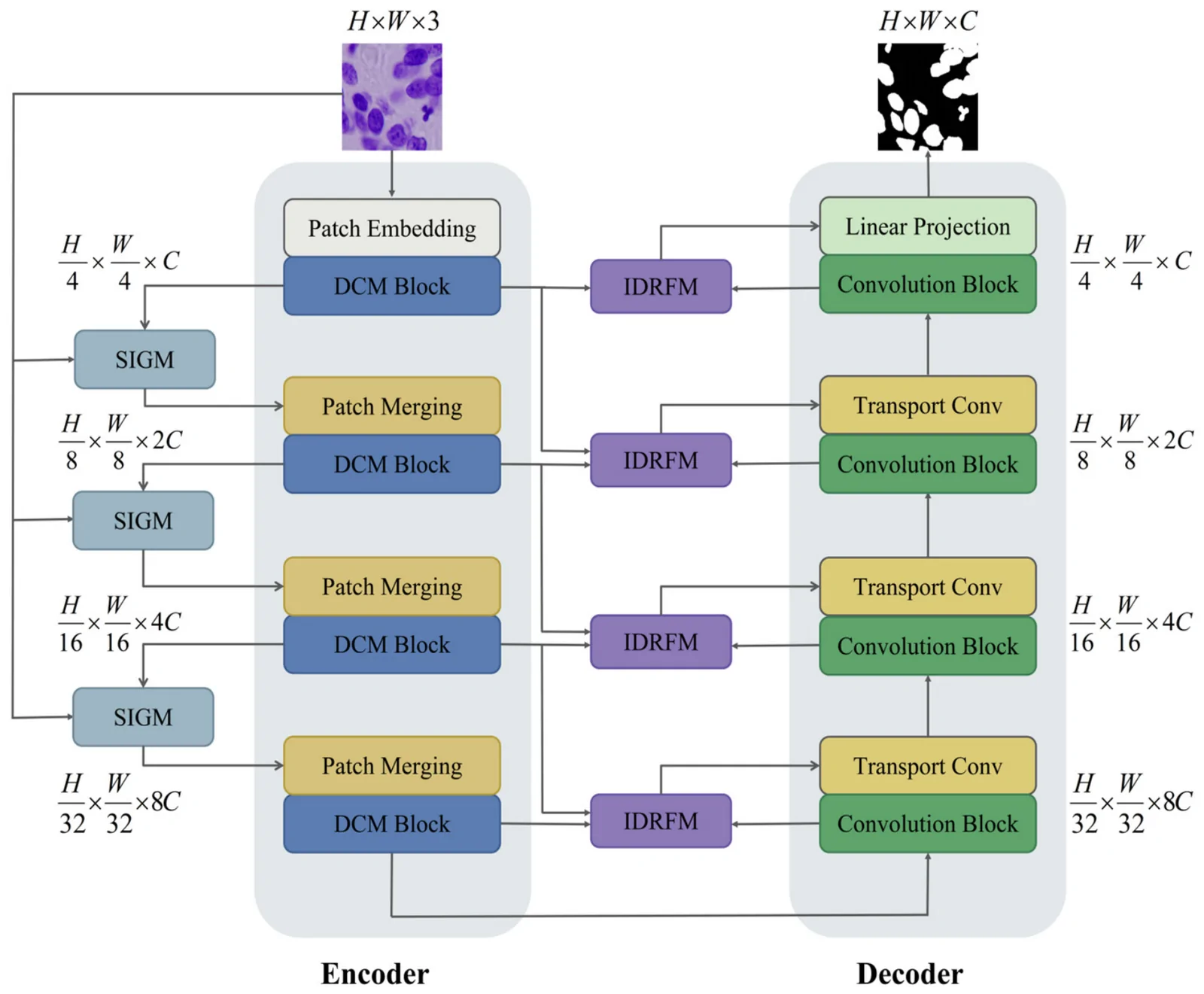
Overall architecture of DIG-MambaNet. The encoder uses DCM Blocks to model local details and global context, SIGM introduces source-image guidance during encoding, and IDRFM improves feature fusion in the decoder for detail recovery and segmentation prediction.

As shown in Figure 1, DIG-MambaNet adopts an encoder–decoder architecture consisting of a patch embedding layer, a hierarchical encoder with DCM Blocks, SIGM-based source image guidance, IDRFM-based feature fusion, and a lightweight decoder. Given an input medical image $I\in R^{\left(H\times W\times 3\right)}$, the network first converts it into an initial feature representation through patch embedding. Specifically, the input image is divided into non-overlapping 4×4 patches, and each patch is projected to a feature dimension of C, where C denotes the base channel number and is set to 64 by default. Layer Normalization (LN) is then applied to obtain the initial feature map $F_{0}\in R^{H/4\times W/4\times C}$, which is fed into the hierarchical encoder.

The initial feature map is processed by a four-stage hierarchical encoder for multi-level feature extraction. Each stage uses a DCM Block to model local fine-grained textures and global semantic information in parallel, followed by adaptive interaction and fusion of the two feature streams. From the second stage onward, patch merging is first applied to reduce the spatial resolution and increase the channel dimension before the features enter the DCM Block. This design progressively halves the spatial resolution and doubles the channel dimension, producing encoder feature maps with spatial resolutions of [H/4,H/8,H/16,H/32] and corresponding channel dimensions of [C,2C,4C,8C].

To mitigate the loss of fine spatial structures caused by consecutive downsampling, SIGM is introduced in the first three encoder stages. At each stage, SIGM uses the scale-aligned original image as spatial guidance to extract high-frequency details. These details are injected into the corresponding feature maps through a gating mechanism, strengthening boundary and structural representations during deep semantic modeling.

In the decoding stage, a lightweight four-stage decoder is used for feature recovery. Following MPFC-Net [38], the decoder adopts a composite module to refine and reorganize features. In the last three stages, transposed convolutions progressively upsample the feature maps, increasing the spatial resolution while halving the channel dimension at each stage. To improve cross-layer feature fusion, IDRFM is embedded into the decoding process. At each upsampling stage, IDRFM takes the current decoder feature and the encoder feature at the same spatial resolution as inputs, and further uses the higher-resolution encoder feature from the previous stage as structural guidance. This design improves cross-layer alignment during reconstruction. After the final fusion stage, the decoder produces a complete feature map of size H/4×W/4×C, which is converted into the segmentation prediction by the final projection layer.

### 3.2. Dual-Path Complementary Modeling Block

Accurate medical image segmentation requires both fine-grained local detail modeling and global semantic understanding. Convolutional operations are effective for capturing textures and boundaries, but their long-range dependency modeling ability is limited. By contrast, Mamba-based state space modeling efficiently captures global context but is less effective at preserving local high-frequency details. To combine these complementary strengths, we design a dual-path complementary modeling block (DCM Block), which contains parallel CNN and Mamba branches for local and global feature extraction, respectively. A cross-feature spatial interaction module (CSIM) is further introduced to adaptively fuse the two feature streams.

**Figure 2:**
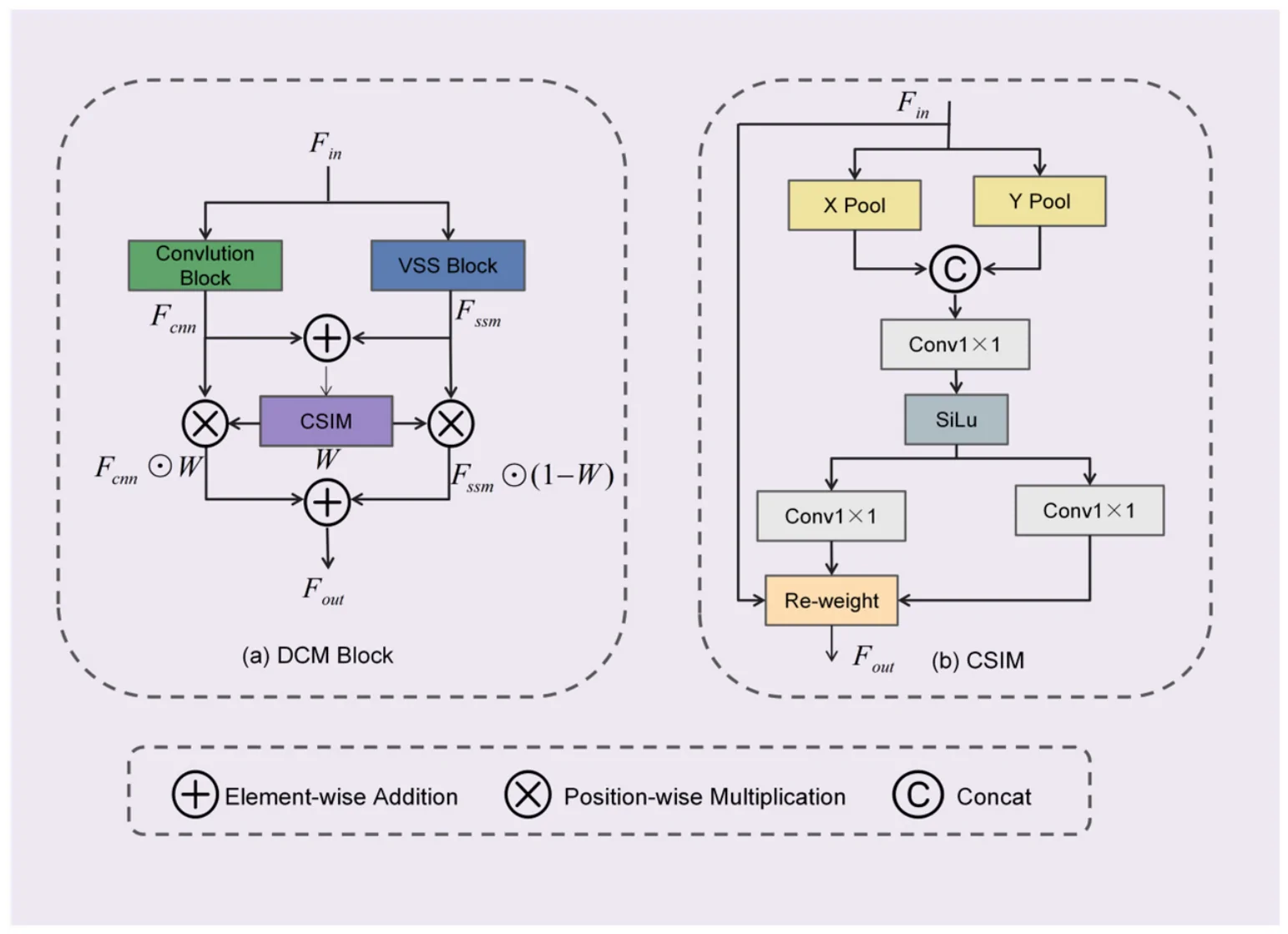
Structure of the DCM Block and the internal CSIM module.

As shown in Figure 2, given an input feature map $F_{in}\in R^{B\times C\times H\times W}$, the DCM Block first duplicates the input and sends it to two parallel branches. The CNN branch focuses on local high-frequency textures and boundary responses. It consists of a standard residual convolutional block with two sets of “3 × 3 Conv + BN + SiLU” layers, together with a residual connection to improve training stability:

(1) $$F_{cnn}=F_{cnn}(F_{in})=BN_{2}\left(Conv_{3\times 3}\left(\sigma \left(BN_{1}\left(Conv_{3\times 3}(F_{in})\right)\right)\right)\right)+F_{in}$$

where $\sigma \left(\cdot \right)$ denotes the SiLU activation function. The Mamba branch uses a two-dimensional Visual State Space (VSS) Block with four-direction selective scanning to model long-range dependencies over the spatial feature grid. Following the standard formulation of discrete state space models, the scanning process can be expressed as:

(2) $$h_{t}=Ah_{t-1}+Bx_{t}$$

(3) $$y_{t}=Ch_{t}$$

where $x_{t}$ is the input sequence unfolded according to the scanning order, $h_{t}$ is the hidden state, $y_{t}$ is the output sequence, and A,B,C are learnable parameters of the state space model. The outputs from four-direction scanning are aggregated and reshaped into spatial features to obtain the global representation:

(4) $$F_{\mathrm{ssm}}=F_{\mathrm{vss}}(F_{\mathrm{in}})+F_{\mathrm{in}}$$

To model the relative contributions of the local feature $F_{cnn}$ and the global feature $F_{\mathrm{ssm}}$ at different spatial locations, CSIM generates a spatially adaptive gating map W for feature fusion. First, the two branch features are preliminarily aggregated to form a unified receptive representation:

(5) $$F_{\mathrm{mid}}=F_{\mathrm{cnn}}+F_{\mathrm{ssm}}$$

Inspired by CBAM [24], CSIM adopts both average and max pooling to capture complementary feature responses. Different from general spatial attention, CSIM performs direction-aware pooling along the height and width directions to preserve spatial positional cues. Specifically, average and max pooling are applied to $F_{\mathrm{mid}}$ along both the height and width directions:

(6) $$X_{h}=\mathrm{Concat}([{\mathrm{AvgPool}}_{W}(F_{\mathrm{mid}}),\mathrm{MaxPoo}l_{W}(F_{\mathrm{mid}})],\dim=1)\in R^{B\times 2C\times H\times 1}$$

(7) $$X_{w}=\mathrm{Concat}([{\mathrm{AvgPool}}_{H}(F_{\mathrm{mid}}),\mathrm{MaxPoo}l_{H}(F_{\mathrm{mid}})],\dim=1)\in R^{B\times 2C\times 1\times W}$$

Then, $X_{w}$ is transposed and concatenated with $X_{h}$ along the spatial dimension, followed by a shared 1 × 1 convolution for dimensional reduction:

(8) $$X_{\mathrm{cat}}=\mathrm{Concat}([X_{h},X_{w}^{T}],\dim=2)\in R^{B\times 2C\times (H+W)\times 1}$$

(9) $$X_{\mathrm{mid}}=\mathrm{SiLU}({\mathrm{Conv}}_{1\times 1}(X_{\mathrm{cat}}))\in R^{B\times \frac{C}{r}\times \left(H+W\right)\times 1}$$

Subsequently, $X_{\mathrm{mid}}$ is split along the spatial dimension into $X_{h}^{\mathrm{mid}}\in R^{B\times C/r\times H\times 1}$ and $X_{w}^{\mathrm{mid}}\in R^{B\times C/r\times 1\times W}$. Each branch is passed through an independent 1 × 1 convolution and a Sigmoid function to generate directional gating weights, where r denotes the channel reduction ratio, which is used to control the intermediate channel dimension. In all experiments, we set r = 16.

(10) $$g_{h}=\sigma_{s}({\mathrm{Conv}}_{1\times 1}(X_{h}^{\mathrm{mid}}))\in R^{B\times C\times H\times 1}$$

(11) $$g_{w}=\sigma_{s}({\mathrm{Conv}}_{1\times 1}(X_{w}^{\mathrm{mid}}))\in R^{B\times C\times 1\times W}$$

The final spatially adaptive gating map is obtained by element-wise multiplication:

(12) $$W=g_{h}⊙g_{w}\in R^{B\times C\times H\times W}$$

This directional gating design is related to Coordinate Attention [27], but CSIM uses both average and max pooling along each spatial direction to capture complementary responses and generates a spatially adaptive gating map to control the relative contributions of the CNN branch and the Mamba branch during local-global feature fusion. Using the dynamic weighting map W, adaptive fusion is performed as follows:

(13) $$F_{\mathrm{out}}=F_{\mathrm{cnn}}⊙W+F_{\mathrm{ssm}}⊙(1-W)$$

This fusion strategy enables spatially adaptive integration of local and global representations. In texture-rich or boundary-sensitive regions, the network can assign greater weight to CNN features, whereas in relatively homogeneous regions it can rely more on Mamba features to improve global consistency. Thus, the DCM Block balances local detail preservation and long-range dependency modeling with limited additional computational cost.

### 3.3. Source Image-Guided Module (SIGM)

**Figure 3:**
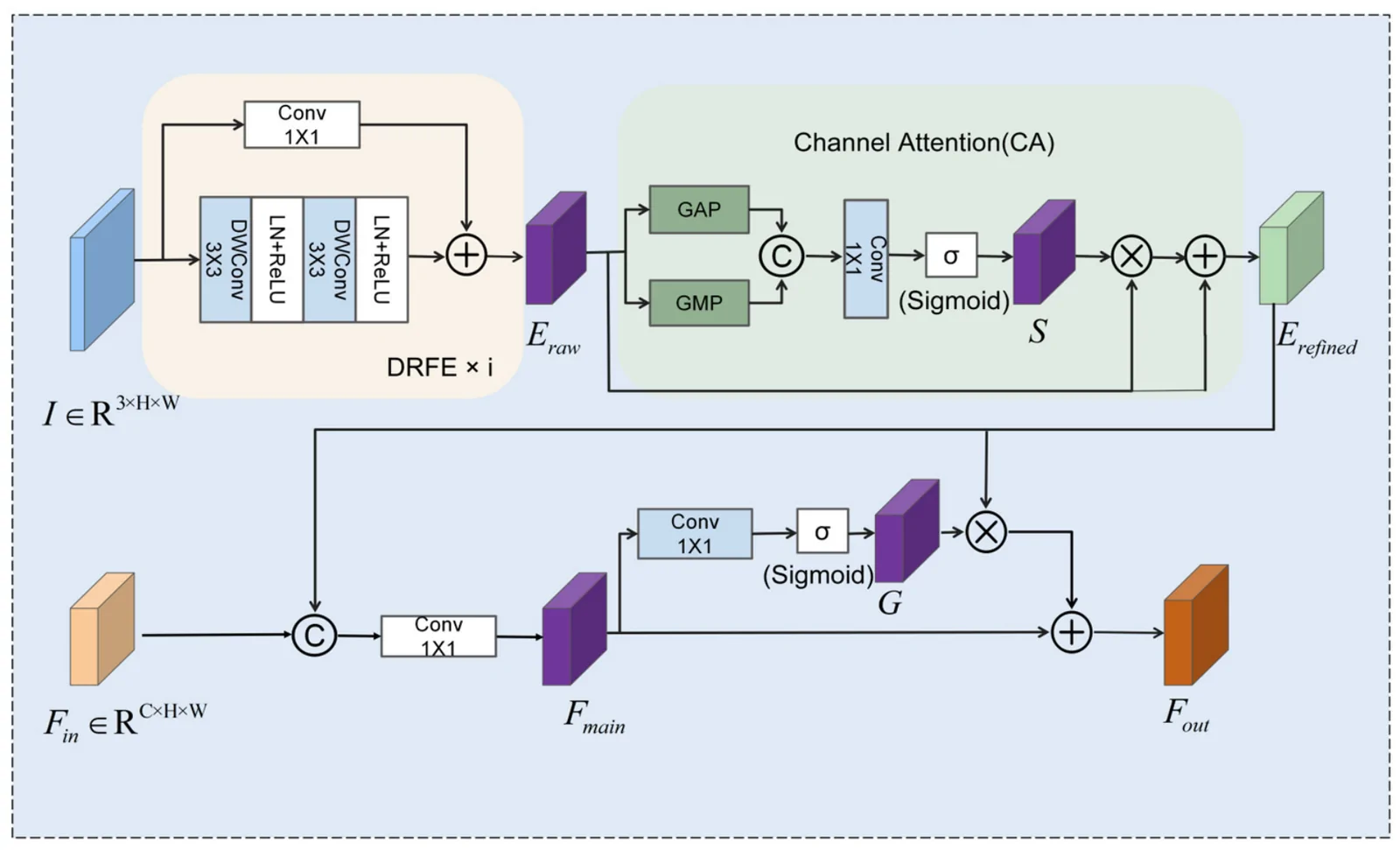
The architecture of the Source Image-Guided Module (SIGM).

In medical image segmentation, repeated downsampling enlarges the receptive field and improves semantic abstraction, but it also weakens source-level boundary and texture cues from the original image. The loss of such fine structural information can cause blurred contours and incomplete lesion or anatomical region delineation, especially in images with low contrast, weak boundaries, or complex appearance variations. To address this issue, we design the source image-guided module (SIGM), as shown in Figure 3. SIGM extracts fine-grained details from the source image and injects them into deep feature representations through a gating mechanism. For a given encoder stage, let the input feature map be $F_{\mathrm{in}}\in R^{B\times C\times H\times W}$ and the source image be $I_{\mathrm{raw}}\in R^{B\times 3\times H_{0}\times W_{0}}$.

First, the source image is resized to match the spatial resolution of the current feature map:

(14) $$I=\mathrm{Resize}(I_{\mathrm{raw}})\in R^{B\times 3\times H\times W}$$

Next, a 1 × 1 convolution projects the resized input into the channel space C, producing $I_{\mathrm{proj}}$:

(15) $$I_{\mathrm{proj}}={\mathrm{Conv}}_{1\times 1}(I)\in R^{B\times C\times H\times W}$$

To extract details aligned with the current feature resolution, we introduce a Detail Refinement Feature Extraction Block (DRFE Block). In the first three encoder stages, one DRFE Block is used at each stage, and the numbers of DRFE layers are explicitly set to N1 = 1, N2 = 2, and N3 = 3, respectively. Each DRFE layer contains two successive depthwise separable convolutions with normalization and nonlinear activation, which enhances detail modeling while keeping the parameter count and computational cost low.

(16) $$F_{\mathrm{temp}}^{\left(k\right)}=\mathrm{ReLU}(\mathrm{LN}({\mathrm{DWConv}}_{3\times 3}(F_{d}^{\left(k-1\right)})))$$

(17) $$F_{d}^{\left(k\right)}=\mathrm{ReLU}(\mathrm{LN}({\mathrm{DWConv}}_{3\times 3}(F_{\mathrm{temp}}^{\left(k\right)})))\;k=1,2,\ldots ,N_{l}$$

After $N_{l}$ iterations, the extracted detail features are fused with the projected input through residual addition:

(18) $$E_{\mathrm{raw}}=I_{\mathrm{proj}}+F_{d}^{\left(N_{l}\right)}\in R^{B\times C\times H\times W}$$

To suppress potential background noise in high-frequency image details, SIGM applies channel attention to $E_{\mathrm{raw}}$. Global average pooling (GAP) and global max pooling (GMP) are first performed along the spatial dimensions:

(19) $$V=\mathrm{Concat}(\mathrm{GAP}(E_{\mathrm{raw}}),\mathrm{GMP}(E_{\mathrm{raw}}))\in R^{B\times 2C\times 1\times 1}$$

Two consecutive 1 × 1 convolutions are then used to generate channel-wise weights.

(20) $$S=\sigma_{s}({\mathrm{Conv}}_{1\times 1}^{2}(\mathrm{ReLU}({\mathrm{Conv}}_{1\times 1}^{1}(V))))\in R^{B\times C\times 1\times 1}$$

where $\sigma_{s}(\cdot )$ is the Sigmoid function. The final refined feature map is obtained by channel-wise multiplication:

(21) $$E_{\mathrm{refined}}=E_{\mathrm{raw}}⊙S\in R^{B\times C\times H\times W}$$

After detail refinement, SIGM concatenates $E_{\mathrm{refined}}$ with the main feature map $F_{\mathrm{in}}$ along the channel dimension and applies a 1 × 1 convolution for adaptive injection:

(22) $$F_{\mathrm{main}}={\mathrm{Conv}}_{1\times 1}(\mathrm{Concat}(F_{\mathrm{in}},E_{\mathrm{refined}}))\in R^{B\times C\times H\times W}$$

Finally, a gating map G is predicted to control the contribution of source-guided features in both spatial and channel dimensions:

(23) $$G=\sigma_{s}({\mathrm{Conv}}_{1\times 1}(F_{\mathrm{main}}))\in R^{B\times C\times H\times W}$$

The output of SIGM is obtained through residual selective injection:

(24) $$F_{\mathrm{out}}=F_{\mathrm{main}}+G⊙E_{\mathrm{refined}}\in R^{B\times C\times H\times W}$$

In this way, SIGM adaptively injects source-guided detail cues into deep feature maps, improving boundary preservation and structural representation, particularly in regions with weak contrast or complex appearance variations.

### 3.4. Inter-Layer Detail Refinement Fusion Module (IDRFM)

**Figure 4:**
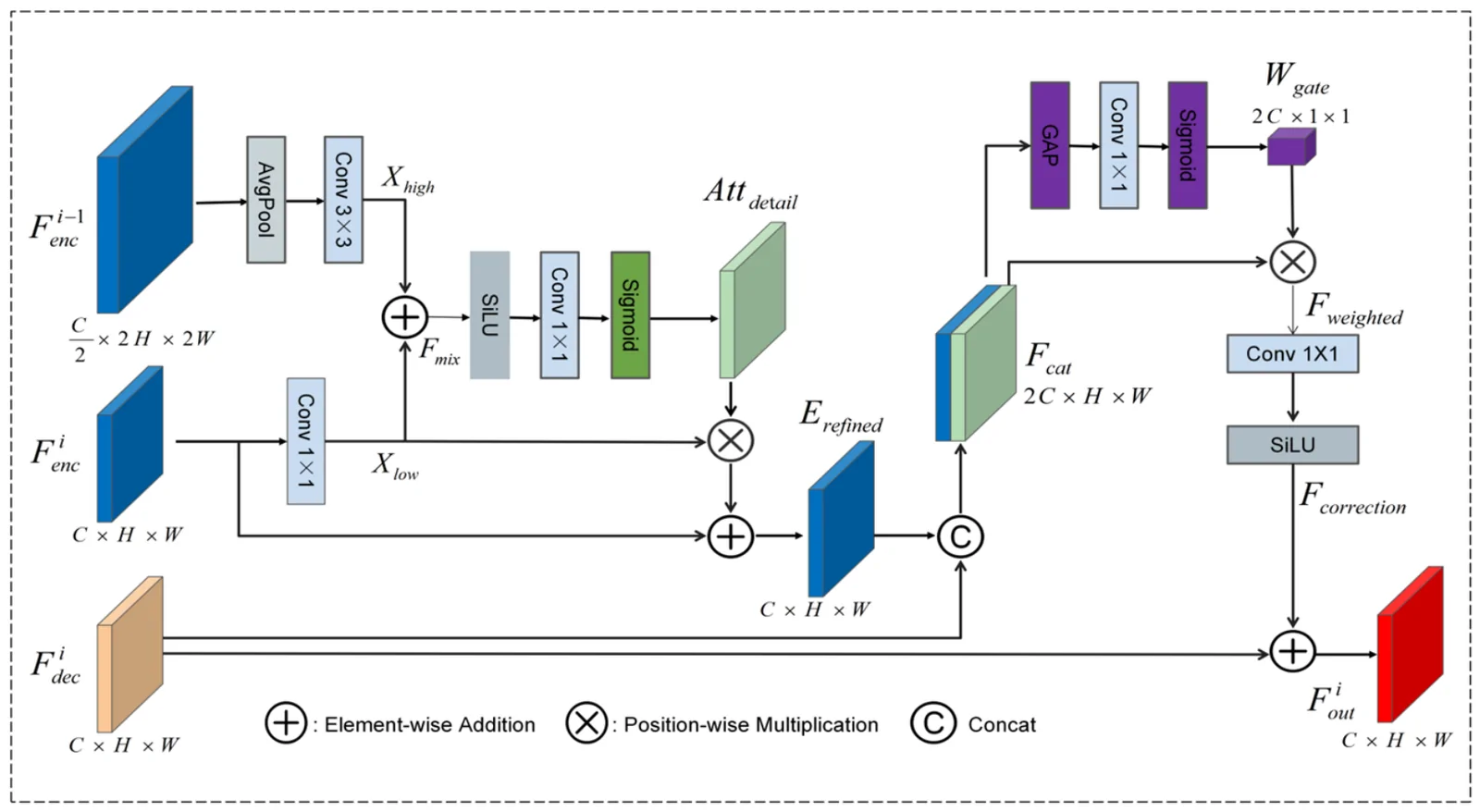
Structure of the Inter-layer Detail Refinement Fusion Module (IDRFM).

During decoding, effective fusion of encoder and decoder features is critical for recovering structural continuity and boundary details. Conventional skip connections based on direct concatenation or addition often ignore the mismatch between deep semantic representations and shallow spatial details, resulting in insufficient feature alignment. To address this problem, we propose the inter-layer detail refinement fusion module (IDRFM), as shown in Figure 4. IDRFM first refines encoder features using higher-resolution structural guidance and then adaptively fuses the refined representation with decoder features.

Let the decoder feature at the i-th decoding stage be $F_{dec}^{i}\in R^{B\times C\times H\times W}$, the encoder feature at the same resolution be $F_{\mathrm{enc}}^{i}\in R^{B\times C\times H\times W}$, and the encoder feature from the previous higher-resolution stage be $F_{enc}^{i-1}\in R^{\left(B\times C/2\times 2H\times 2W\right)}$. IDRFM uses the richer structural details in $F_{enc}^{i-1}$ to refine the current encoder feature $F_{enc}^{i}$, and then fuses the refined encoder representation with the decoder feature $F_{dec}^{i}$ to generate the output. Since the spatial resolution of $F_{enc}^{i-1}$ is 2H × 2W, spatial alignment is first performed using 2 × 2 average pooling with a stride of 2, followed by a 3 × 3 convolution:

(25) $$X_{\mathrm{high}}={\mathrm{Conv}}_{3\times 3}({\mathrm{AvgPool}}_{k=2,s=2}(F_{\mathrm{enc}}^{i-1}))\in R^{B\times C\times H\times W}$$

The convolution maps the channel dimension from C/2 to C and further refines the aligned features through local context aggregation. Meanwhile, the encoder feature at the current scale is projected using a 1 × 1 convolution:

(26) $$X_{\mathrm{low}}={\mathrm{Conv}}_{1\times 1}(F_{\mathrm{enc}}^{i})\in R^{B\times C\times H\times W}$$

The two aligned features are then combined to produce an integrated representation:

(27) $$F_{\mathrm{mix}}=X_{\mathrm{high}}+X_{\mathrm{low}}$$

A lightweight nonlinear transformation and a 1 × 1 convolution are subsequently used to generate a spatially adaptive attention map, followed by Sigmoid activation to obtain the gating weights:

(28) $$Att_{\mathrm{detail}}=\sigma_{s}({\mathrm{Conv}}_{1\times 1}(\mathrm{SiLU}(F_{\mathrm{mix}})))\in R^{B\times C\times H\times W}$$

where $\sigma_{s}(\cdot )$ denotes the Sigmoid function. The attention map enhances the encoder features through residual refinement:

(29) $$E_{\mathrm{refined}}=F_{\mathrm{enc}}^{i}+(F_{\mathrm{enc}}^{i}⊙Att_{\mathrm{detail}})\in R^{B\times C\times H\times W}$$

This residual refinement preserves the base representation of $F_{enc}^{i}$ while emphasizing texture and boundary cues in regions with strong attention responses. Thus, structural priors from the higher-resolution encoder stage are transferred to the current scale. After the refined encoder feature $E_{\mathrm{refined}}$ is obtained, it is concatenated with the decoder feature $F_{dec}^{i}$ along the channel dimension:

(30) $$F_{cat}=\mathrm{Concat}(E_{refined},F_{dec}^{i})\in R^{B\times 2C\times H\times W}$$

To maintain computational efficiency, IDRFM uses a lightweight channel gating scheme to model the global importance of the concatenated features. Global average pooling (GAP) is first applied to $F_{cat}$, followed by a 1 × 1 convolution and Sigmoid activation:

(31) $$W_{gate}=\sigma_{s}({\mathrm{Conv}}_{1\times 1}(\mathrm{GAP}(F_{cat})))\in R^{B\times 2C\times 1\times 1}$$

This lightweight design reduces additional computational overhead. The concatenated feature is then reweighted:

(32) $$F_{weighted}=F_{cat}⊙W_{gate}\in R^{B\times 2C\times H\times W}$$

Subsequently, a 1 × 1 convolution projects the features back to C channels, followed by SiLU activation to generate a correction term for the decoder features:

(33) $$F_{correction}=\mathrm{SiLU}({\mathrm{Conv}}_{1\times 1}(F_{weighted}))\in R^{B\times C\times H\times W}$$

Finally, the refined feature is added to the decoder feature through a residual connection:

(34) $$F_{out}^{i}=F_{dec}^{i}+F_{correction}\in R^{B\times C\times H\times W}$$

Through this design, IDRFM introduces higher-resolution structural guidance into current-scale encoder features and adaptively injects the refined information into the decoder stream. This improves cross-layer feature alignment and enhances boundary recovery during feature reconstruction.

### 3.5. Loss Function

To support both pixel-level classification accuracy and global structural consistency, we adopt Pixel Position Aware (PPA) loss [39] as the training objective. PPA loss consists of weighted BCE (wBCE) loss and weighted IoU (wIoU) loss. The wBCE term improves pixel-wise classification stability, whereas the wIoU term constrains the overall shape and continuity of the predicted mask from a region-overlap perspective. More details can be found in [40,41]. The weighted IoU loss is defined as:

(35) $$L_{wIoU}=1-\frac{\sum_{i=1}^{H}\sum_{j=1}^{W}w_{ij}G_{ij}P_{ij}}{\sum_{i=1}^{H}\sum_{j=1}^{W}w_{ij}(G_{ij}+P_{ij}-G_{ij}P_{ij})}$$

where $G_{ij}$ and $P_{ij}$ denote the ground truth label and predicted probability at pixel (i,j), respectively. This term enhances structural consistency by assigning higher weights to overlapping regions. The weighted BCE loss is defined as:

(36) $$L_{wBCE}=-\frac{\sum_{i=1}^{H}\sum_{j=1}^{W}w_{ij}[G_{ij}\log(P_{ij})+(1-G_{ij})\log(1-P_{ij})]}{\sum_{i=1}^{H}\sum_{j=1}^{W}w_{ij}}$$

This term enforces pixel-level consistency between predictions and ground truth, while the weighting factor $w_{ij}$ emphasizes critical regions such as boundaries and fine structures. Finally, the overall PPA loss is defined as:

(37) $$L=L_{wIoU}+L_{wBCE}$$

## 4. Experiments

This section describes the experimental protocol, including the datasets, evaluation metrics, implementation settings, comparative experiments, ablation studies, and discussion of model behavior.

### 4.1. Datasets

To evaluate the segmentation performance and robustness of DIG-MambaNet under diverse medical imaging conditions, we conduct experiments on four representative datasets: 2018DSB [42], ISIC2018 [43], JSUAH-Cerebellum [44], and CVC-ClinicDB [45]. These datasets represent nuclei segmentation in microscopy, skin lesion segmentation in dermoscopy, fetal cerebellum segmentation in ultrasound, and polyp segmentation in colonoscopy, respectively. They encompass diverse target structures, object scales, boundary characteristics, noise patterns, and background complexities, providing complementary conditions for assessing local detail preservation, global structural modeling, boundary delineation, and performance across modality-specific imaging challenges.

The 2018DSB dataset [42], derived from the Data Science Bowl 2018 nuclei segmentation challenge, contains cell images acquired under diverse microscopic imaging conditions. Accurate nuclei segmentation is important for quantitative bioimage analysis, including cell counting and morphological characterization. Nuclei are often small and densely distributed, and adjacent instances may adhere to or overlap with one another. Their boundaries can be indistinct, and background appearance varies with staining and imaging conditions. These characteristics make accurate separation of neighboring nuclei difficult and require models to capture fine local details together with contextual relationships.

The ISIC2018 dataset [43], released by the International Skin Imaging Collaboration for skin lesion analysis, is a widely used benchmark for medical image segmentation. Accurate lesion delineation can support quantitative assessment of lesion size, shape, and border characteristics in computer-assisted dermoscopic analysis. Lesion regions often show ambiguous boundaries, low contrast, large shape variations, and heterogeneous internal textures. Hair occlusion, illumination variation, and imaging artifacts may further interfere with segmentation. Under these conditions, preserving lesion integrity and boundary continuity requires coordinated modeling of local details and global structure.

The JSUAH-Cerebellum dataset [44] is used for fetal cerebellum segmentation in ultrasound imaging. Reliable delineation of the fetal cerebellum can support quantitative assessment of cerebellar morphology and development in prenatal ultrasound. Compared with optical images, ultrasound images usually contain strong speckle noise, low contrast, and blurred boundaries. Anatomical appearance may also vary with imaging angle, individual differences, and gestational age. These factors make robust segmentation difficult and require the model to maintain global structural consistency under noisy imaging conditions.

The CVC-ClinicDB dataset [45] is commonly used for polyp segmentation in colonoscopy images. It contains 612 fully annotated polyp images collected from colonoscopy video sequences. Accurate polyp localization and boundary delineation are relevant to computer-assisted colonoscopy and quantitative lesion assessment. Polyps vary substantially in size, shape, and appearance. Low contrast between the lesion and surrounding mucosa, specular highlights, vascular patterns, and complex background textures can interfere with accurate segmentation. Therefore, effective polyp segmentation requires preservation of subtle local structures while using contextual information to distinguish lesions from surrounding tissue and imaging artifacts.

The number of training, validation, and test samples for the four datasets is summarized in Table 1. The JSUAH-Cerebellum split follows the experimental protocol used in previous studies [6,8].

### 4.2. Evaluation Metrics

To quantitatively evaluate segmentation performance, we adopt five commonly used metrics: Dice, IoU, Precision, Recall, and Accuracy. These metrics assess complementary aspects of segmentation quality, including region overlap, foreground discrimination, and overall pixel-wise correctness. Dice measures the overlap between the predicted segmentation and the ground truth, with higher values indicating better segmentation performance. IoU evaluates the ratio between the intersection and union of the predicted and ground-truth regions. Precision measures the proportion of correctly predicted positive pixels among all predicted positives, whereas Recall measures the proportion of correctly predicted positive pixels among all ground-truth positives. Accuracy measures the proportion of correctly classified pixels over the entire image.

The formulations of these metrics are defined as follows:

(38) $$Dice=\frac{2\left|GT\cap Pre\right|}{\left|GT\right|+\left|Pre\right|}$$

(39) $$IoU=\frac{\left|GT\cap Pre\right|}{\left|GT\cup Pre\right|}$$

(40) $$Precision=\frac{TP}{TP+FP}$$

(41) $$Recall=\frac{TP}{TP+FN}$$

(42) $$Acc=\frac{TP+TN}{TP+TN+FP+FN}$$

where Pre denotes the predicted segmentation result and GT denotes the ground truth. TP and TN represent correctly predicted foreground and background pixels, respectively, while FP and FN denote background pixels incorrectly classified as foreground and foreground pixels incorrectly classified as background, respectively.

### 4.3. Implementation Details

All experiments are conducted on a workstation equipped with an NVIDIA RTX 4090D GPU with 24 GB memory. The software environment includes Ubuntu 22.04, Python 3.10, PyTorch 2.1.0, CUDA 12.1, and cuDNN with default configurations. For training, all models are optimized using the Adam optimizer with an initial learning rate of $1\times 10^{-4}$. A cosine learning rate schedule is used for dynamic adjustment, and all models are trained for 400 epochs. To ensure a fair comparison, the input images and corresponding ground-truth masks from all datasets are resized to 256×256. Data augmentation, including random rotation, horizontal flipping, and vertical flipping, is applied during training to improve robustness to spatial variations and morphological changes. Specifically, images were resized using bilinear interpolation, whereas ground-truth masks were resized using nearest-neighbor interpolation to preserve discrete label values. This unified square-resizing protocol was applied to all compared models, and its effect on CVC-ClinicDB was further examined through an input-resolution sensitivity analysis.

### 4.4. Experimental Results and Analysis

This section reports and analyzes the experimental results of DIG-MambaNet on the 2018DSB, ISIC2018, JSUAH-Cerebellum, and CVC-ClinicDB datasets. The proposed method is compared with representative CNN-based, Transformer-based, Mamba-based, and hybrid segmentation models, including U-Net [2], Attention U-Net [4], SE-Net [23], TransUNet [11], UCTransNet [28], VM-UNet [33], AC-MambaSeg [46], H-vmunet [35], UltraLight VM-UNet [36], MFEVM-UNet [47], and nnU-Net [48]. Both quantitative and qualitative analyses are performed. Quantitative comparisons evaluate standard segmentation metrics, whereas qualitative visualizations examine segmentation quality in challenging regions such as ambiguous boundaries, noisy areas, and complex structures. These experiments are designed to assess the effectiveness of DIG-MambaNet in local detail preservation, global structural modeling, and robust segmentation across diverse medical imaging scenarios.

#### 4.4.1. Quantitative Evaluation

In this subsection, DIG-MambaNet is compared with representative segmentation methods across different medical imaging scenarios. Table 2, Table 3, Table 4 and Table 5 summarize the quantitative results on the 2018DSB, ISIC2018, JSUAH-Cerebellum, and CVC-ClinicDB datasets, with the best and second-best results highlighted in bold and underlined, respectively.

On the 2018DSB dataset, as shown in Table 2, DIG-MambaNet achieves the best mIoU, mDice, mRecall, and mAcc, reaching 88.15%, 93.05%, 94.34%, and 98.06%, respectively. Compared with U-Net, it improves mIoU and mDice by 0.84 and 1.46 percentage points, respectively. It also shows competitive performance compared with Mamba-based or hybrid methods such as AC-MambaSeg and H-vmunet in terms of mIoU and mDice. However, its mPrecision is 92.73%, which is lower than that of U-Net and SE-Net by 0.43 and 0.78 percentage points, respectively. This result suggests that DIG-MambaNet tends to produce more inclusive foreground predictions on 2018DSB. Since nuclei in this dataset are often small, densely distributed, and adjacent or partially overlapping, improving the coverage of nuclear regions can increase recall and region-level overlap, but may also include a small number of background pixels near weak or touching boundaries. Therefore, the lower mPrecision reflects a mild increase in false-positive responses, whereas the higher mRecall, mIoU, and mDice suggest that DIG-MambaNet is effective in preserving nuclear completeness and modeling contextual relationships among neighboring targets. This overall behavior is consistent with the role of the DCM Block and CSIM in strengthening the complementary fusion between local structural details and broader contextual information.

On the ISIC2018 dataset, DIG-MambaNet benefits from the coordinated modeling of local details, global context, and cross-layer feature refinement, which improves lesion representation under complex dermoscopic conditions. As shown in Table 3, DIG-MambaNet achieves the best mDice, mPrecision, and mAcc among all compared methods, reaching 92.59%, 94.72%, and 96.39%, respectively. Compared with Attention U-Net, it improves mDice and mPrecision by 1.55 and 2.21 percentage points, respectively, and it also outperforms VM-UNet in these core metrics. Its mIoU is 86.78%, which is slightly lower than that of TransUNet (87.20%) and H-vmunet (87.24%), with small margins of 0.42 and 0.46 percentage points, respectively, and its mRecall is lower than several competing methods. This discrepancy indicates that DIG-MambaNet tends to produce more precise and compact lesion predictions, as reflected by the highest mPrecision, while being relatively conservative in covering some ambiguous peripheral lesion regions. Such behavior is consistent with the challenging characteristics of ISIC2018, including irregular lesion shapes, blurred boundaries, low contrast, and heterogeneous textures. Therefore, DIG-MambaNet achieves a favorable overall trade-off by obtaining the highest mDice, mPrecision, and mAcc while maintaining competitive mIoU performance.

On the JSUAH-Cerebellum dataset, DIG-MambaNet demonstrates strong performance under ultrasound imaging conditions characterized by speckle noise, low contrast, and blurred anatomical boundaries. As reported in Table 4, it achieves the best mIoU, mDice, mPrecision, and mAcc among all compared methods. Compared with UCTransNet, DIG-MambaNet improves mIoU and mDice by 1.26 and 0.87 percentage points, respectively, indicating more accurate structural delineation. It also performs better than MFEVM-UNet on most core metrics. These results suggest that DIG-MambaNet is effective at preserving global structural consistency while reducing the effect of noise interference, which is important for reliable ultrasound segmentation.

On the CVC-ClinicDB dataset, as shown in Table 5, DIG-MambaNet achieves the best mIoU and mDice among all compared methods, indicating strong segmentation capability in complex colonoscopy scenes. Compared with TransUNet, it improves mIoU and mDice by 1.39 and 1.13 percentage points, respectively, and the corresponding gains over H-vmunet are 1.95 and 1.44 percentage points. These improvements are consistent with the coordinated effects of CSIM, SIGM, and IDRFM. For polyp segmentation with subtle boundaries, specular reflections, and complex mucosal textures, CSIM enhances local-global feature interaction, SIGM provides source-level boundary guidance, and IDRFM improves structural recovery during decoding. As a result, DIG-MambaNet achieves more accurate polyp delineation in challenging endoscopic images.

**Figure 5:**
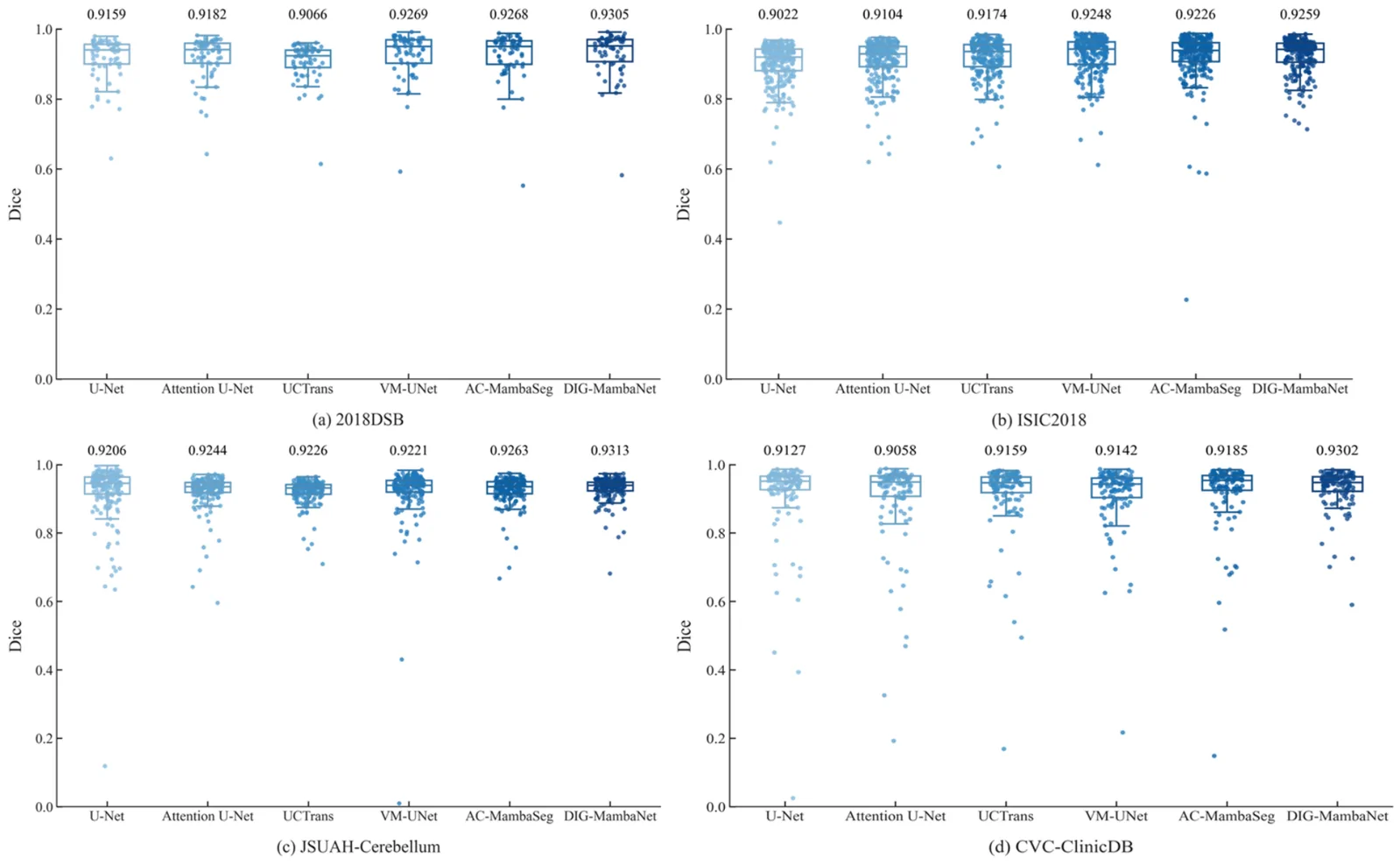
Boxplots of per-image Dice scores for different segmentation models on four datasets: (**a**) 2018DSB, (**b**) ISIC2018, (**c**) JSUAH-Cerebellum, and (**d**) CVC-ClinicDB. Boxes indicate the interquartile range, whiskers extend to 1.5× IQR, and scatter points represent individual samples. Values above the boxes denote mean Dice scores.

To further assess stability and robustness, Figure 5 presents boxplots of per-image Dice scores on the four datasets. Compared with the average results reported in Table 2, Table 3, Table 4 and Table 5, the boxplots provide a more detailed view of performance variation across individual samples. DIG-MambaNet consistently maintains a higher median Dice score across all datasets, indicating stronger overall segmentation quality. Its interquartile range is relatively compact, suggesting lower variability across test samples. On challenging datasets such as ISIC2018 and CVC-ClinicDB, DIG-MambaNet also shows fewer low-score cases, reflecting improved robustness under ambiguous boundaries, complex textures, and background interference. On 2018DSB and JSUAH-Cerebellum, the Dice distributions are concentrated in the high-score range, further supporting its reliability in dense microscopic scenes and noisy ultrasound images.

Overall, results across the four datasets show that DIG-MambaNet delivers accurate, stable, and balanced segmentation under diverse medical imaging conditions. Improvements in core metrics and favorable per-image Dice distributions indicate effective integration of local detail preservation, global context modeling, and cross-layer refinement, supporting consistent performance on tasks with complex structures, ambiguous boundaries, and heterogeneous image characteristics.

#### 4.4.2. Qualitative Evaluation

**Figure 6:**
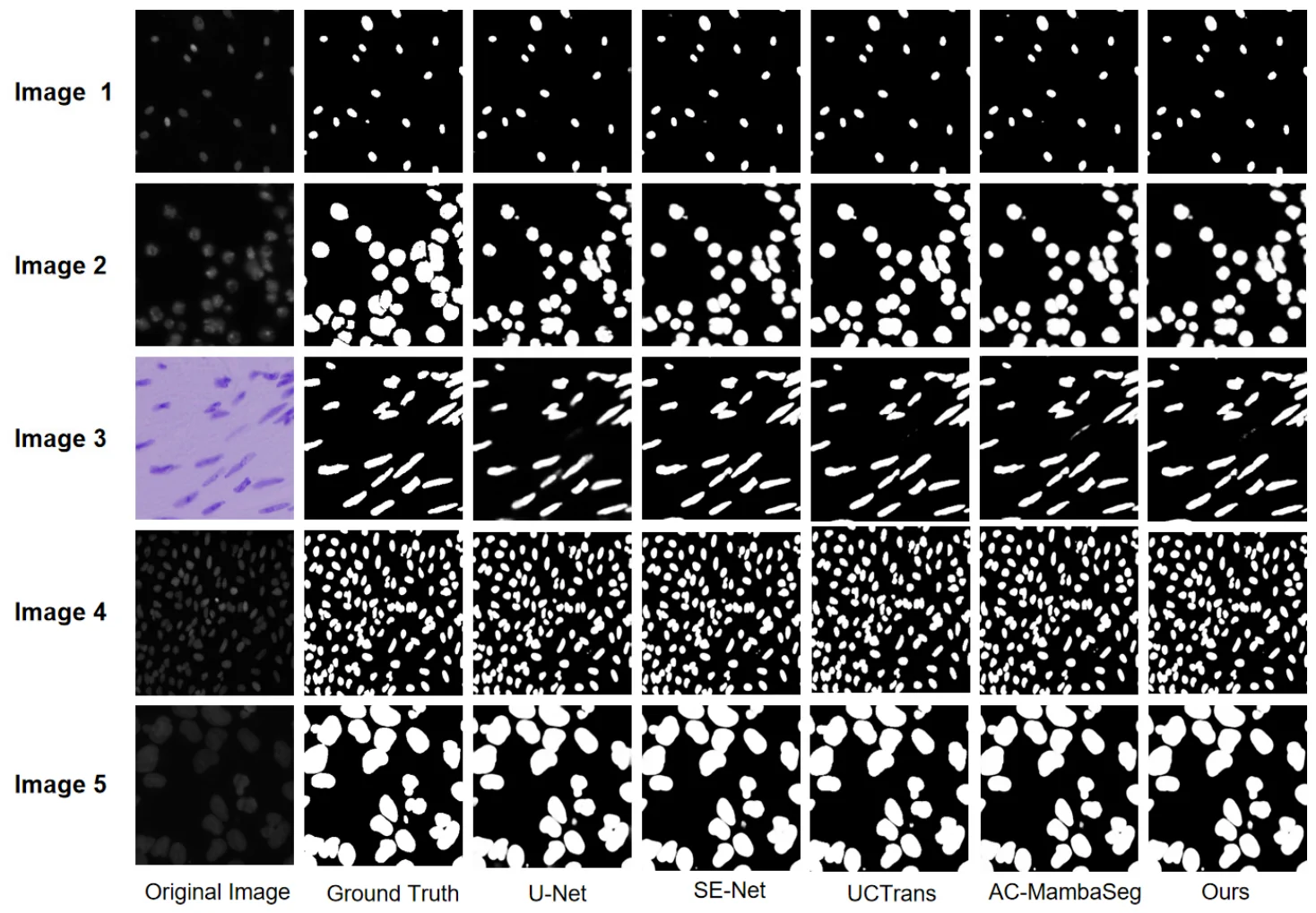
Segmentation results of different models on the 2018DSB dataset. Image 1 indicates a sparse scene containing small, low-contrast nuclei; Image 2 indicates a crowded scene containing densely clustered and touching nuclei; Image 3 indicates elongated, spindle-shaped nuclei; Image 4 indicates numerous densely distributed small nuclei; and Image 5 indicates large, closely spaced nuclei with low contrast and blurred boundaries.

**Figure 7:**
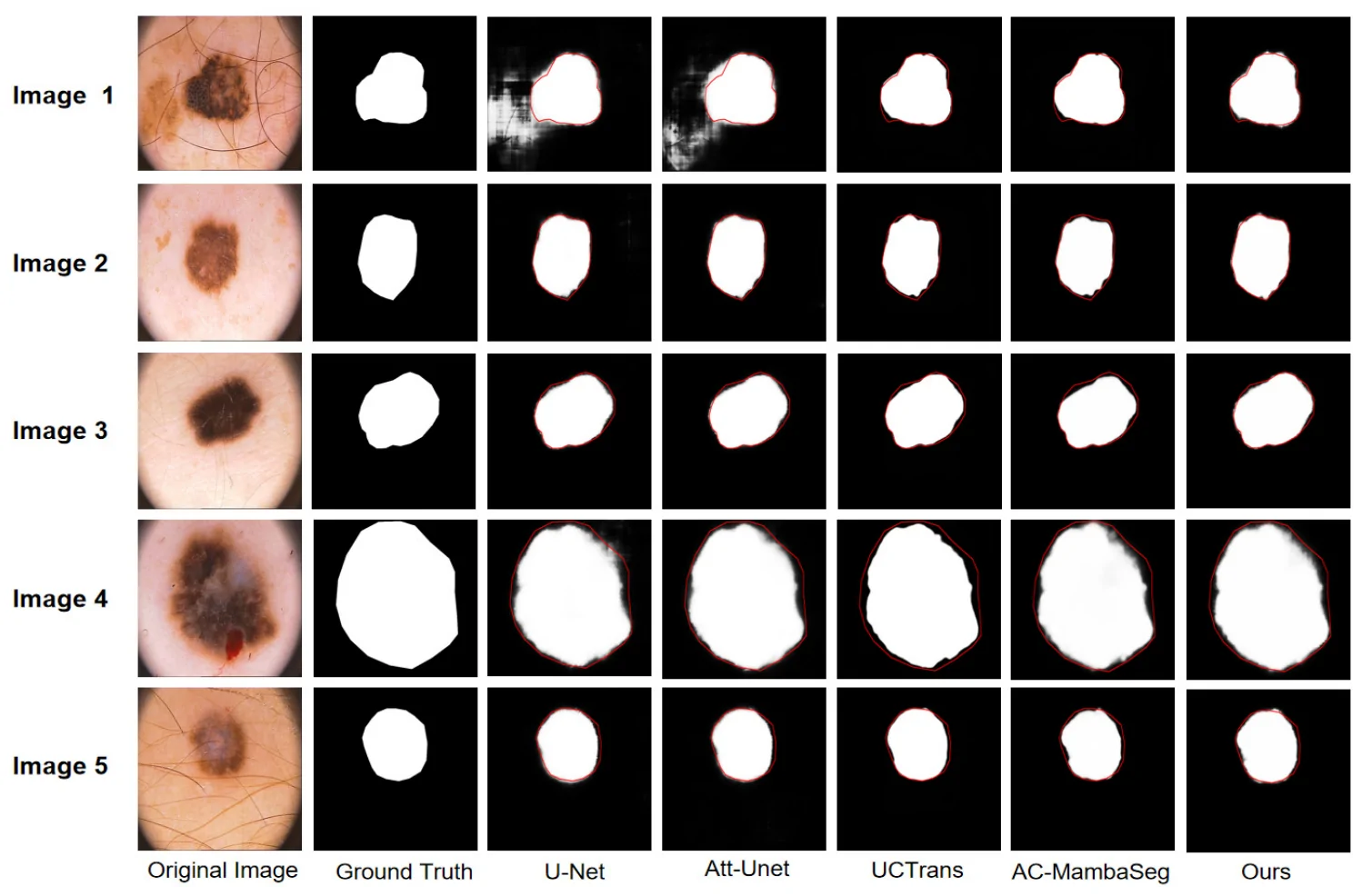
Segmentation results of different models on the ISIC2018 dataset. Image 1 indicates an irregularly shaped dark lesion affected by hair occlusion; Image 2 indicates a vertically elongated lesion with a relatively smooth and well-defined boundary; Image 3 indicates a compact dark lesion with a relatively regular contour; Image 4 indicates a large lesion with a heterogeneous internal appearance and an indistinct, irregular boundary; and Image 5 indicates a small, low-contrast lesion affected by hair occlusion.

**Figure 8:**
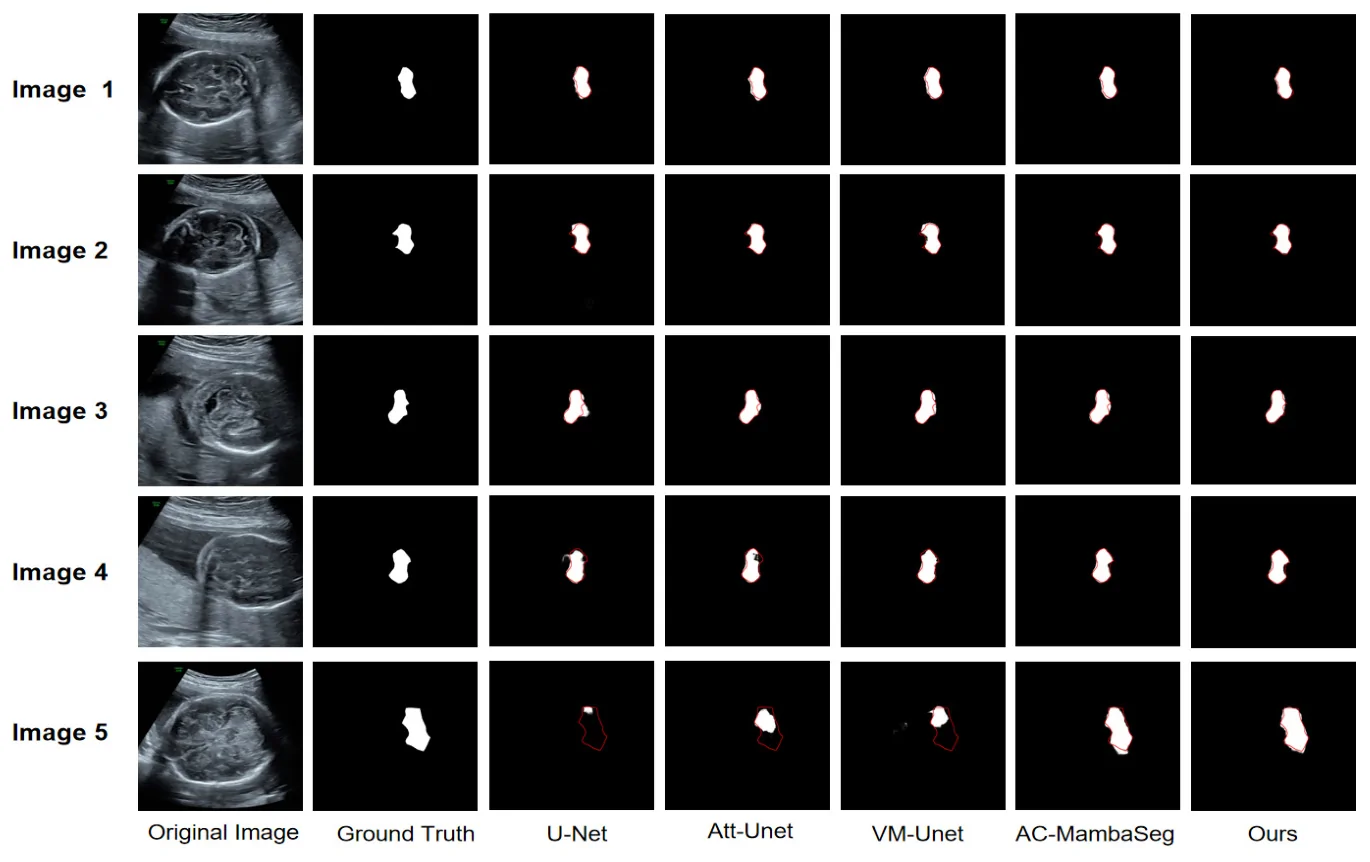
Segmentation results of different models on the JSUAH-Cerebellum dataset. Image 1 indicates a small, vertically oriented cerebellar region; Image 2 indicates a compact cerebellar region with an irregular shape; Image 3 indicates an elongated and curved cerebellar region with indistinct boundaries; Image 4 indicates a small cerebellar region surrounded by heterogeneous ultrasound texture; and Image 5 indicates a relatively large, elongated cerebellar region with substantial boundary ambiguity.

**Figure 9:**
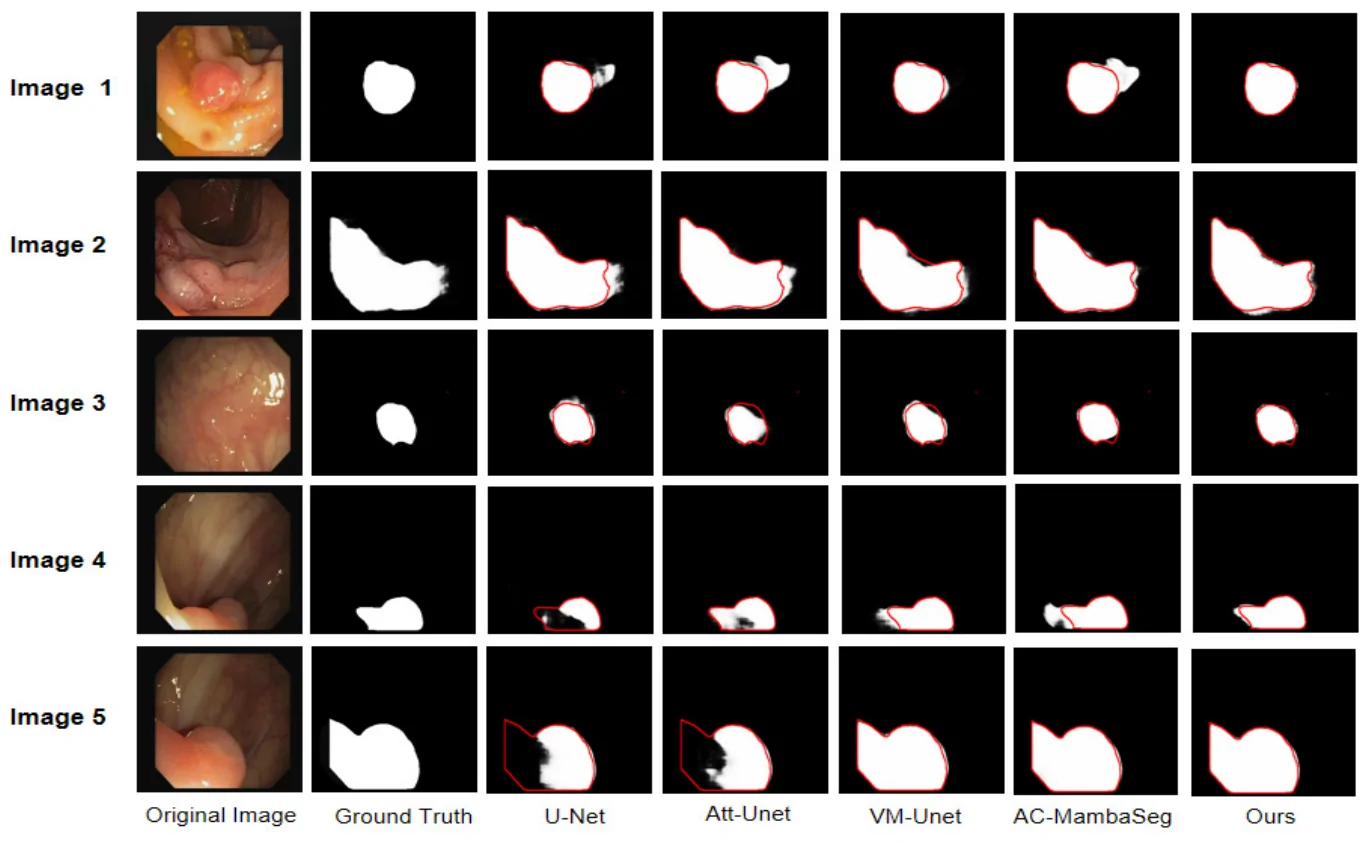
Segmentation results of different models on the CVC-ClinicDB dataset. Image 1 indicates a protruding polyp affected by specular reflections and surrounding mucosal textures; Image 2 indicates a large, irregular polyp extending along the lower image boundary amid complex mucosal folds; Image 3 indicates a small, low-contrast polyp; Image 4 indicates a small, partially visible polyp located near the lower image boundary against a complex background; and Image 5 indicates a large, partially visible polyp with an irregular contour extending beyond the image boundary.

To provide a more intuitive comparison, qualitative results on representative samples from 2018DSB, ISIC2018, JSUAH-Cerebellum, and CVC-ClinicDB are shown in Figure 6, Figure 7, Figure 8 and Figure 9. In each figure, the first column shows the input image, the second column shows the ground truth, and the last column shows the prediction of DIG-MambaNet, while the remaining columns present the results of comparison methods.

Figure 6 compares different methods in densely distributed nuclei scenes from the 2018DSB dataset. Traditional CNN-based methods, such as U-Net and SE-Net, tend to merge adjacent nuclei and fail to recover complete boundaries in crowded regions, resulting in insufficient instance separation. Although UCTransNet and AC-MambaSeg produce relatively stable overall contours, they still show boundary shifts and local shape distortions in small targets or low-contrast regions. In contrast, DIG-MambaNet generates contours that more closely match the ground truth and preserves clearer separation between adjacent nuclei, reducing false positives caused by background interference.

Figure 7 presents qualitative results for skin lesion segmentation. In Image 1, U-Net shows over-segmentation, with predicted regions extending beyond the ground-truth boundary. Attention U-Net and UCTransNet improve contour stability, but boundary deviations remain. DIG-MambaNet generates predictions that are more consistent with the ground truth and better aligned with lesion boundaries. In Image 4, where lesion shapes are irregular and background textures are complex, several comparison methods produce blurred contours or local boundary shifts. DIG-MambaNet preserves more complete and smoother lesion boundaries, indicating better lesion morphology preservation under complex texture conditions.

Figure 8 shows the results for ultrasound cerebellum segmentation. In Image 5, U-Net produces severe false negatives and predicts only small regions. Attention U-Net roughly localizes the target but still underestimates the region. VM-UNet and AC-MambaSeg maintain more stable contours but exhibit local boundary deviations. DIG-MambaNet achieves more accurate localization while preserving structural integrity and natural shape, even for blurred or elongated structures. In Image 3, where the target has an elongated shape and indistinct boundaries, competing methods still show contour deviations, whereas DIG-MambaNet maintains better structural consistency. These observations suggest robust performance under low-contrast and noisy ultrasound conditions.

Figure 9 illustrates polyp segmentation results on CVC-ClinicDB. In Image 1, U-Net and Attention U-Net show boundary deviations and redundant responses outside the lesion. VM-UNet and AC-MambaSeg improve overall contour accuracy, but local over-segmentation and incomplete boundary fitting remain. DIG-MambaNet produces predictions more closely aligned with the ground truth, with cleaner contours and reduced background interference. In Image 4, where the polyp is small and the surrounding background is complex, several comparison methods show unstable localization or incomplete target recovery, whereas DIG-MambaNet better preserves the lesion shape and suppresses irrelevant background activation. Similar trends can also be observed in Images 2 and 5.

Overall, the qualitative results across the four datasets show that DIG-MambaNet produces predictions that are more consistent with the ground truth in dense microscopic scenes, ambiguous lesions, noisy ultrasound images, and complex endoscopic backgrounds. These visual comparisons further support its advantages in boundary delineation, fine structure recovery, and suppression of irrelevant interference.

**Figure 10:**
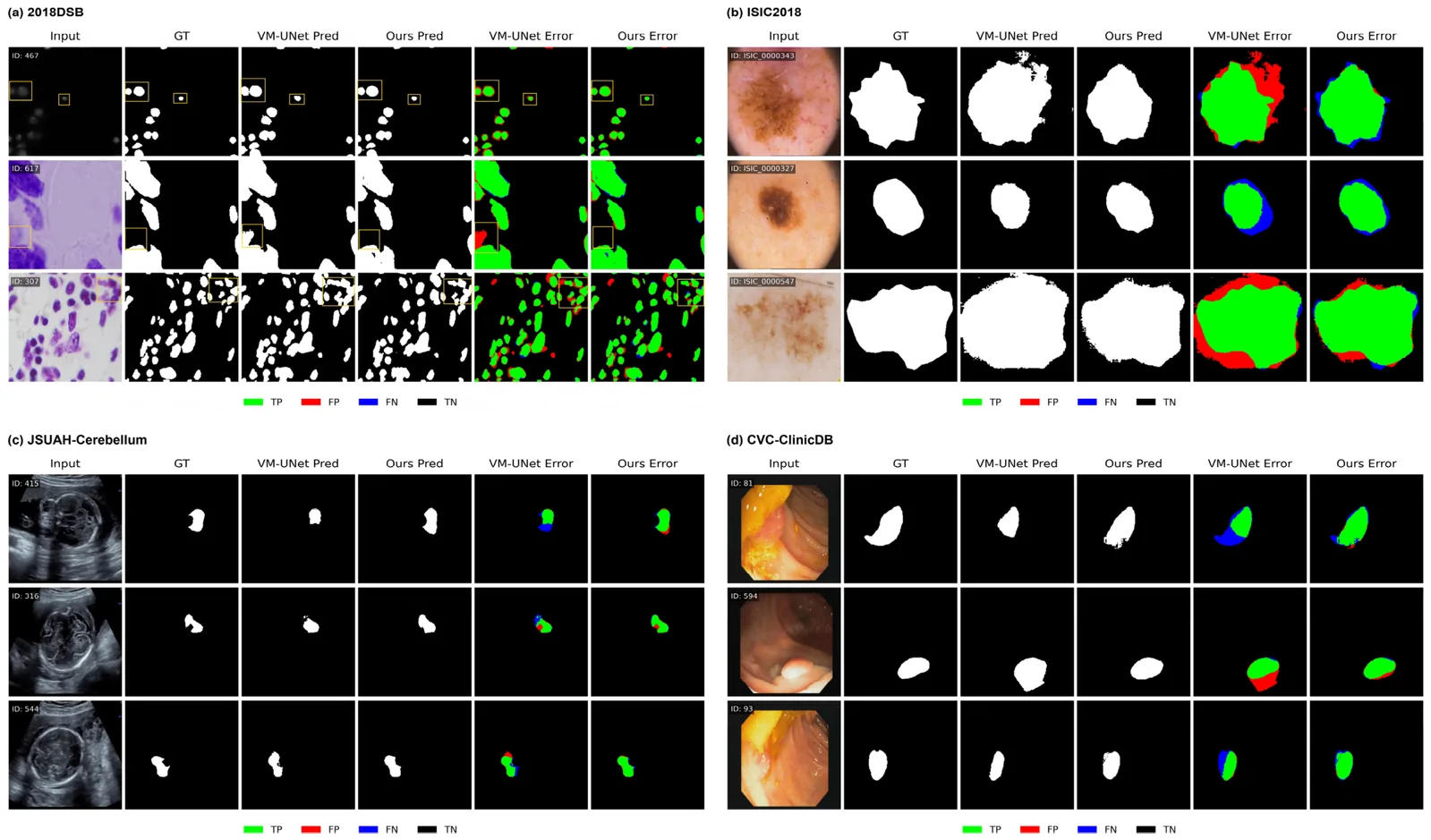
Error-map comparison between VM-UNet and DIG-MambaNet on four datasets: (**a**) 2018DSB, (**b**) ISIC2018, (**c**) JSUAH-Cerebellum and (**d**) CVC-ClinicDB. Green, red, blue, and black indicate true-positive, false-positive, false-negative, and true-negative pixels, respectively.

To further clarify the visual differences among model predictions, we selected VM-UNet and DIG-MambaNet for error-map comparison in Figure 10. In these maps, true positive, false positive, false negative, and true negative pixels are shown in green, red, blue, and black, respectively, allowing the spatial distribution of segmentation errors to be directly inspected. Across the four datasets, DIG-MambaNet produced fewer false-positive and false-negative regions than VM-UNet. On 2018DSB, DIG-MambaNet better separated densely distributed nuclei and reduced local background interference. On ISIC2018, it suppressed redundant responses around lesion boundaries and generated more compact lesion regions. For JSUAH-Cerebellum, the proposed method reduced missed target regions under low-contrast ultrasound conditions, indicating more accurate localization of ambiguous anatomical structures. On CVC-ClinicDB, DIG-MambaNet showed fewer boundary-related errors and less background activation in small or irregular polyp regions. These error-map comparisons provide explicit visual evidence that DIG-MambaNet improves boundary delineation and reduces both over-segmentation and under-segmentation compared with VM-UNet.

#### 4.4.3. Efficiency Analysis

Practical medical image segmentation requires a careful balance between segmentation accuracy and computational efficiency, especially when models are expected to be deployed in clinical or resource-constrained environments. To address this issue, we further evaluated the computational complexity and inference efficiency of DIG-MambaNet and representative baseline models under the same input size of 256 × 256. As summarized in Table 6, the comparison includes mDice, trainable parameters, FLOPs, single-image inference time, FPS, and peak VRAM usage. All measurements of inference time, FPS, and peak VRAM were obtained on an NVIDIA GeForce RTX 4090D GPU with a batch size of 1 under the same evaluation protocol.

Standard convolutional baselines show limited accuracy–efficiency balance despite their relatively mature encoder–decoder designs. U-Net requires 54.738 G FLOPs while achieving an mDice of 91.27%, which is 1.75 percentage points lower than DIG-MambaNet. Attention U-Net further increases the computational cost to 66.632 G FLOPs but obtains an mDice of 90.58%, trailing our method by 2.44 percentage points. Although SE-Net reduces the model scale to 7.821 M parameters and 13.754 G FLOPs through channel recalibration, its mDice remains 91.85%, suggesting that lightweight channel attention alone may be insufficient to achieve the same accuracy level on this dataset. nnU-Net achieves a stronger mDice of 92.42%, but it requires 115.786 G FLOPs, representing more than 13 times the computational footprint of DIG-MambaNet. Conversely, Transformer-based architectures such as TransUNet and UCTransNet introduce global context modeling through attention mechanisms, but this comes with substantial parameter redundancy and computational overhead. TransUNet contains 105.322 M parameters and 32.229 G FLOPs, while UCTransNet contains 66.488 M parameters and 43.058 G FLOPs. In contrast, DIG-MambaNet requires only 25.054 M parameters and 8.910 G FLOPs, while achieving a higher mDice of 93.02%.

The emergence of State Space Models provides a more efficient path for long-range dependency modeling. Among Mamba-based models, UltraLight VM-UNet achieves the lowest model complexity, with only 0.049 M parameters and 0.065 G FLOPs, but its mDice is 89.24%, which is 3.78 percentage points lower than that of DIG-MambaNet. Similarly, H-vmunet requires only 0.742 G FLOPs, but its mDice remains 91.58%, trailing DIG-MambaNet by 1.44 percentage points. These results suggest that reducing parameters and FLOPs alone does not necessarily lead to a better accuracy–efficiency trade-off, especially for polyp segmentation where subtle boundaries, irregular shapes, and complex background textures require sufficient feature representation capacity.

The trade-off between performance gains and complexity is particularly evident when comparing DIG-MambaNet with the stronger hybrid Mamba-based baseline MFEVM-UNet. MFEVM-UNet obtains an mDice of 92.25%, but it requires 61.747 M parameters, 12.665 G FLOPs, and 35.709 ms/img inference time. In contrast, DIG-MambaNet improves mDice to 93.02% while reducing the parameter count to 25.054 M, FLOPs to 8.910 G, and inference time to 22.917 ms/img. This balance is mainly attributed to the coordinated design of DCM, CSIM, SIGM, and IDRFM, which enables the network to integrate CNN-based local details, Mamba-based global dependencies, source-image-guided high-frequency cues, and cross-layer refinement without excessive model expansion. Therefore, DIG-MambaNet achieves a more favorable balance between segmentation accuracy, computational complexity, and inference efficiency.

#### 4.4.4. Ablation Study

**Figure 11:**
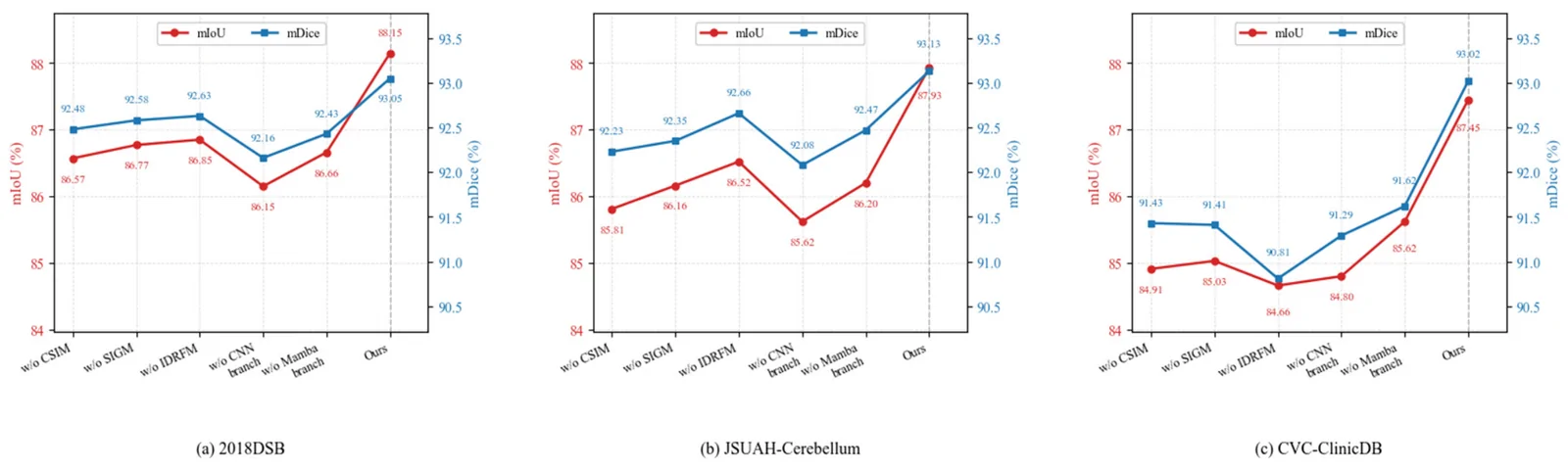
Ablation performance trends of DIG-MambaNet on three datasets. The red and blue curves denote mIoU and mDice, respectively.

To evaluate the contribution of each key component in DIG-MambaNet, ablation experiments are conducted on 2018DSB, JSUAH-Cerebellum, and CVC-ClinicDB. Table 7, Table 8 and Table 9 report the quantitative results under different module configurations, and Figure 11 summarizes the trends of mIoU and mDice across the three datasets. In addition, Table 10 provides an attention replacement ablation study on the JSUAH-Cerebellum dataset to compare CSIM with representative attention mechanisms. Except for architectural variations, all experiments use the same data split, input setting, and training strategy to ensure fair comparison.

(1) Effectiveness of the DCM Block.

To examine the necessity of dual-path complementary modeling, we separately remove the CNN branch and the Mamba branch from the DCM Block. As shown in Table 7, Table 8 and Table 9 and Figure 11, removing either branch leads to consistent performance degradation, confirming that local convolutional features and global Mamba representations are complementary. On 2018DSB, removing the CNN branch reduces mIoU and mDice from 88.15 and 93.05 to 86.15 and 92.16, respectively, indicating that global context modeling alone is insufficient for preserving fine-grained nuclear structures. Removing the Mamba branch also decreases mIoU and mDice to 86.66 and 92.43, suggesting that local convolution alone cannot fully capture global semantic relationships. This effect is more evident on CVC-ClinicDB, where the full model improves mDice by 1.73 and 1.40 percentage points over the variants without the CNN branch and without the Mamba branch, respectively. These results show that both branches are necessary for maintaining a balance between boundary-sensitive local representation and global contextual modeling.

To further evaluate the role of CSIM, we replace it with a conventional concatenation-based fusion operation, where the CNN and Mamba branch features are concatenated and then projected by a 1 × 1 convolution. As reported in Table 7, Table 8 and Table 9 and visualized in Figure 11, the *w*/*o* CSIM variant consistently performs worse than the full model. The degradation is particularly clear on JSUAH-Cerebellum and CVC-ClinicDB, where removing CSIM decreases mDice by 0.90 and 1.59 percentage points, respectively. These results indicate that simple concatenation-based fusion is less effective than CSIM in exploiting the complementarity between CNN-based local features and Mamba-based global features. The larger performance drops on ultrasound and colonoscopy images further suggest that spatially adaptive local-global interaction is useful under noisy imaging conditions and complex endoscopic backgrounds.

(2) Effectiveness of the SIGM.

As shown in Table 7, Table 8 and Table 9 and Figure 11, removing the Source Image-Guided Module (SIGM) consistently degrades segmentation performance across the three datasets. On 2018DSB and JSUAH-Cerebellum, the *w*/*o* SIGM variant decreases mIoU by 1.38 and 1.77 percentage points, and decreases mDice by 0.47 and 0.78 percentage points, respectively. A more pronounced degradation is observed on CVC-ClinicDB, where removing SIGM decreases mIoU and mDice from 87.45 and 93.02 to 85.03 and 91.41, respectively. These results indicate that source-image-guided detail compensation contributes to segmentation accuracy across different imaging modalities. By introducing high-frequency cues from the original image into deep feature maps, SIGM helps alleviate the loss of boundary and texture information caused by progressive downsampling, thereby improving the representation of fine structures and ambiguous contours.

(3) Effectiveness of the IDRFM.

IDRFM is designed to refine encoder–decoder feature fusion by using higher-resolution structural information to guide current-scale feature reconstruction. To verify its contribution, we replace IDRFM with conventional skip connections. As shown in Table 7, Table 8 and Table 9 and Figure 11, this replacement leads to clear performance degradation on all three datasets. The mIoU decreases by 1.30, 1.41, and 2.29 percentage points on 2018DSB, JSUAH-Cerebellum, and CVC-ClinicDB, respectively, while the corresponding mDice decreases are 0.42, 0.47, and 1.21 percentage points. These results indicate that direct skip fusion cannot fully resolve the semantic and spatial mismatch between encoder and decoder features. In contrast, IDRFM improves cross-layer feature alignment and supports the recovery of structural details during decoding, which contributes to more accurate segmentation across different imaging modalities.

(4) Comparison with representative attention mechanisms.

To further examine the effectiveness of CSIM, we replace CSIM with representative attention mechanisms, including SE [23], CBAM [24], and Coordinate Attention [27], on the JSUAH-Cerebellum dataset, while keeping the remaining architecture, data split, and training strategy unchanged. As shown in Table 10, all attention-based replacement variants outperform the variant without CSIM, indicating that attention-based feature modulation is beneficial for feature fusion. The original CSIM achieves the best mIoU, mDice, mPrecision, and mAcc, reaching 87.93%, 93.13%, 93.78%, and 99.74%, respectively. Compared with SE, CBAM, and Coordinate Attention, CSIM improves mIoU by 1.18, 1.25, and 0.63 percentage points, and improves mDice by 0.33, 0.35, and 0.30 percentage points, respectively. Although Coordinate Attention obtains the highest mRecall, its lower mPrecision suggests that the improvement in recall may be accompanied by less selective foreground prediction. This may be related to its directional average pooling strategy, which tends to encode smoother and broader positional context and may preserve more foreground pixels, thereby reducing missed detections. In contrast, CSIM combines average and max pooling along both directions, where average pooling provides contextual information and max pooling emphasizes salient responses such as boundaries, strong foreground regions, or small structures. This more selective gating mechanism helps CSIM suppress irrelevant responses and achieve higher mPrecision, mIoU, and mDice. These results suggest that CSIM more effectively regulates the complementary contributions of CNN-based local details and Mamba-based global context in an adaptive manner.

(5) Effect of Input Image Size.

To further examine whether resizing images to 256 × 256 affects the preservation of fine details, we conducted an input-resolution sensitivity analysis on the CVC-ClinicDB dataset, where subtle polyp boundaries and local texture variations are important for accurate segmentation. The input resolution was varied among 224 × 224, 256 × 256, 320 × 320, and 384 × 384, while the remaining experimental settings were kept consistent. As shown in Table 11, reducing the input resolution to 224 × 224 decreases the mIoU and mDice to 86.78% and 92.19%, respectively, indicating that an excessively small resolution may discard useful boundary and structural information. When the input resolution is increased from 256 × 256 to 320 × 320 and 384 × 384, the mIoU slightly improves from 87.45% to 87.64% and 87.75%, respectively. However, the mDice remains nearly unchanged, with 93.02%, 93.00%, and 93.01% obtained at 256 × 256, 320 × 320, and 384 × 384, respectively.

Meanwhile, the computational cost increases substantially with larger input resolutions. In particular, the FLOPs increase from 8.91 G at 256 × 256 to 20.04 G at 384 × 384, whereas the improvement in mIoU is limited to 0.30 percentage points and no further gain is observed in mDice. These results indicate that 256 × 256 provides a reasonable balance between segmentation accuracy and computational efficiency in our experimental setting. Therefore, 256 × 256 was adopted as the default input resolution for the main comparative and ablation experiments.

(6) Effect of the loss function

To further justify the choice of the training objective, we compare PPA loss with three commonly used medical image segmentation losses, including Dice loss, Dice+BCE loss, and Focal loss, on the CVC-ClinicDB dataset. The network architecture and training settings were kept unchanged for all loss functions.

As shown in Table 12, PPA loss achieves the best performance across all evaluated metrics, with 87.45% mIoU, 93.02% mDice, 93.36% mPrecision, 93.46% mRecall, and 98.80% mAcc. Compared with Dice+BCE loss, PPA loss improves mIoU and mDice by 1.41 and 0.69 percentage points, respectively. These results indicate that the combination of weighted BCE and weighted IoU provides more effective supervision for both pixel-level classification and region-level structural consistency.

### 4.5. Discussion

#### 4.5.1. Spatial Modeling Limitations of SSM-Based Architectures and Their Mitigation in DIG-MambaNet

Although DIG-MambaNet achieves strong performance across multiple datasets, vision architectures based on state space models still face a spatial modeling challenge. In Mamba-based vision models, two-dimensional features are usually unfolded into one-dimensional sequences for selective scanning and recursive modeling. This design is effective for long-range dependency modeling, but it may weaken local spatial coherence by disrupting the original neighborhood structure of feature maps. This issue can be more evident in medical images with crowded targets, irregular structures, or ambiguous boundaries, where accurate delineation depends heavily on precise local relationships. The qualitative comparisons in Figure 6, Figure 7, Figure 8 and Figure 9 also show that methods relying more heavily on sequential global modeling tend to produce structural discontinuities or boundary deviations in challenging cases.

DIG-MambaNet introduces several designs to mitigate this limitation and preserve spatial consistency during feature modeling. First, the parallel CNN branch in the DCM Block maintains stable two-dimensional local representations and compensates for the local detail loss associated with the Mamba branch. Second, CSIM supports spatially adaptive interaction between local and global features, allowing the network to balance boundary-sensitive cues and long-range contextual information in different regions. Third, SIGM injects high-frequency information from the source image into deep semantic features, helping recover fine structures weakened by repeated downsampling and sequential modeling. The ablation results in Table 7, Table 8 and Table 9 further support the effectiveness of these components, as performance decreases when the corresponding modules are removed. These findings suggest that preserving spatial coherence is important for accurate medical image segmentation and that hybrid local-global modeling can improve fine-grained structural recovery.

#### 4.5.2. Limitations and Future Work

Although DIG-MambaNet achieves promising results in two-dimensional medical image segmentation, several limitations remain for broader clinical use. First, this study focuses on 2D segmentation, whereas many clinical applications involve three-dimensional volumetric data, such as CT and MRI, which contain richer spatial structures and inter-slice contextual information. Directly extending the current dual-path and multi-module design to 3D data may increase computational complexity and memory consumption. Developing efficient 3D Mamba-based segmentation frameworks is therefore an important direction for future work. Second, although the proposed method is evaluated across multiple imaging modalities, each task uses single-modality input. In clinical practice, multimodal imaging can provide complementary anatomical and functional information, but effective cross-modality feature alignment remains challenging. In addition, pixel-level annotation in medical imaging is expensive and labor-intensive, which limits fully supervised models in low-resource settings and rare disease scenarios. Future work may explore multimodal fusion strategies and self-supervised, semi-supervised, or generative learning methods to better use complementary and unlabeled medical data and improve model generalization.

## 5. Conclusions

In this study, we propose DIG-MambaNet, a Dual-path Interactive Guided Mamba Network for medical image segmentation. The proposed framework integrates CNN-based local detail extraction with the efficient long-range dependency modeling capability of Mamba, aiming to address the persistent difficulty of preserving fine boundaries while maintaining global semantic consistency. The DCM Block with CSIM enables adaptive interaction between local texture cues and global contextual representations, whereas SIGM introduces source-image-guided high-frequency information to compensate for downsampling-induced detail loss. IDRFM further refines cross-layer feature fusion by reducing semantic discrepancies between encoder and decoder features and improving spatial alignment during reconstruction. Experiments on four representative medical image segmentation datasets demonstrate that DIG-MambaNet achieves stable and competitive performance across multiple evaluation metrics, with competitive results in mDice and mIoU and visible improvements in boundary delineation and structural recovery. These findings indicate that coordinated local-global modeling and explicit detail guidance provide an effective strategy for improving segmentation robustness in complex medical images. Future work will explore more efficient extensions to 3D volumetric segmentation, multimodal medical imaging, and learning settings with limited annotations.

## Figures and Tables

**Table 1:** Details of experimental datasets.

Dataset	Total	Train	Valid	Test
2018DSB	670	536	67	67
ISIC2018	2594	2076	259	259
JSUAH-Cerebellum	959	699	68	192
CVC-ClinicDB	612	429	60	123

**Table 2:** Comparison of different models on the 2018DSB dataset.

Model	mIoU	mDice	mPrecision	mRecall	mAcc
U-Net	87.31 ± 0.098	91.59 ± 0.077	93.16 ± 0.146	93.53 ± 0.218	98.01 ± 0.022
Attention U-Net	87.69 ± 0.121	91.82 ± 0.128	93.41 ± 0.210	93.67 ± 0.287	97.91 ± 0.064
SE-Net	87.81 ± 0.190	91.87 ± 0.144	93.51 ± 0.158	93.72 ± 0.207	98.02 ± 0.019
nnU-Net	87.78 ± 0.110	92.45 ± 0.104	93.67 ± 0.152	94.16 ± 0.195	98.03 ± 0.072
TransUNet	87.13 ± 0.142	91.88 ± 0.103	92.73 ± 0.127	93.67 ± 0.094	97.93 ± 0.042
UCTransNet	86.39 ± 0.126	90.66 ± 0.179	93.51 ± 0.231	93.12 ± 0.247	97.88 ± 0.059
VM-UNet	86.96 ± 0.121	92.69 ± 0.168	92.48 ± 0.111	93.73 ± 0.198	97.92 ± 0.034
AC-MambaSeg	87.29 ± 0.109	92.68 ± 0.068	**93.97 ± 0.201**	92.65 ± 0.079	97.98 ± 0.059
H-vmunet	87.74 ± 0.170	92.73 ± 0.144	92.75 ± 0.158	93.19 ± 0.207	97.94 ± 0.019
UltraLight VM-UNet	86.53 ± 0.190	91.25 ± 0.108	91.43 ± 0.158	93.16 ± 0.103	97.55 ± 0.038
MFEVM-UNet	87.83 ± 0.151	92.71 ± 0.168	93.45 ± 0.120	93.76 ± 0.153	98.03 ± 0.046
DIG-MambaNet	**88.15 ± 0.146**	**93.05 ± 0.098**	92.73 ± 0.134	**94.34 ± 0.095**	**98.06 ± 0.069**

**Table 3:** Comparison of different models on the ISIC2018 dataset.

Model	mIoU	mDice	mPrecision	mRecall	mAcc
U-Net	85.15 ± 0.174	90.22 ± 0.099	91.69 ± 0.185	93.66 ± 0.236	95.04 ± 0.088
Attention U-Net	85.91 ± 0.120	91.04 ± 0.139	92.51 ± 0.192	92.81 ± 0.181	95.19 ± 0.059
SE-Net	85.90 ± 0.158	91.36 ± 0.088	91.80 ± 0.134	**93.72 ± 0.171**	96.24 ± 0.051
nnU-Net	86.48 ± 0.112	91.23 ± 0.091	92.94 ± 0.158	92.56 ± 0.137	96.21 ± 0.052
TransUNet	87.20 ± 0.185	91.76 ± 0.190	94.69 ± 0.192	92.43 ± 0.210	96.15 ± 0.075
UCTransNet	85.48 ± 0.185	91.74 ± 0.145	91.86 ± 0.113	92.96 ± 0.159	96.19 ± 0.055
VM-UNet	86.50 ± 0.109	92.48 ± 0.062	93.97 ± 0.222	92.29 ± 0.174	96.27 ± 0.057
AC-MambaSeg	86.25 ± 0.134	92.26 ± 0.086	94.38 ± 0.191	91.61 ± 0.145	96.38 ± 0.043
H-vmunet	**87.24 ± 0.095**	92.42 ± 0.112	94.27 ± 0.154	93.68 ± 0.131	96.17 ± 0.046
UltraLight VM-UNet	83.56 ± 0.097	90.40 ± 0.110	91.97 ± 0.146	90.80 ± 0.133	95.58 ± 0.071
MFEVM-UNet	86.53 ± 0.132	92.37 ± 0.118	94.15 ± 0.159	92.23 ± 0.188	96.32 ± 0.061
DIG-MambaNet	86.78 ± 0.133	**92.59 ± 0.113**	**94.72 ± 0.153**	92.05 ± 0.138	**96.39 ± 0.062**

**Table 4:** Comparison of different models on the JSUAH-Cerebellum dataset.

Model	mIoU	mDice	mPrecision	mRecall	mAcc
U-Net	86.02 ± 0.112	92.06 ± 0.155	91.75 ± 0.130	93.21 ± 0.204	99.71 ± 0.005
Attention U-Net	86.68 ± 0.075	92.44 ± 0.136	92.56 ± 0.200	93.54 ± 0.143	99.72 ± 0.009
SE-Net	87.06 ± 0.098	92.83 ± 0.103	92.94 ± 0.115	93.31 ± 0.176	99.73 ± 0.004
nnU-Net	87.23 ± 0.093	93.06 ± 0.069	93.46 ± 0.098	93.05 ± 0.105	**99.74 ± 0.007**
TransUNet	87.63 ± 0.058	93.06 ± 0.039	93.63 ± 0.131	93.37 ± 0.187	99.73 ± 0.018
UCTransNet	86.67 ± 0.136	92.26 ± 0.168	91.62 ± 0.084	**94.34 ± 0.167**	99.73 ± 0.015
VM-UNet	86.09 ± 0.162	92.21 ± 0.091	91.85 ± 0.175	93.12 ± 0.111	99.64 ± 0.007
AC-MambaSeg	86.51 ± 0.101	92.63 ± 0.194	91.60 ± 0.201	94.10 ± 0.142	99.71 ± 0.012
H-vmunet	86.78 ± 0.168	93.08 ± 0.121	93.12 ± 0.113	93.25 ± 0.189	99.70 ± 0.008
UltraLight VM-UNet	85.42 ± 0.098	91.45 ± 0.106	91.67 ± 0.117	92.50 ± 0.125	99.42 ± 0.006
MFEVM-UNet	87.21 ± 0.129	92.35 ± 0.108	92.97 ± 0.071	94.21 ± 0.141	99.72 ± 0.007
DIG-MambaNet	**87.93 ± 0.124**	**93.13 ± 0.114**	**93.78 ± 0.087**	93.48 ± 0.225	**99.74 ± 0.005**

**Table 5:** Comparison of different models on the CVC-ClinicDB dataset.

Model	mIoU	mDice	mPrecision	mRecall	mAcc
U-Net	85.68 ± 0.153	91.27 ± 0.108	93.09 ± 0.178	92.02 ± 0.203	98.57 ± 0.028
Attention U-Net	84.59 ± 0.130	90.58 ± 0.129	91.52 ± 0.211	91.97 ± 0.178	98.34 ± 0.037
SE-Net	86.45 ± 0.164	91.85 ± 0.093	92.36 ± 0.132	92.48 ± 0.161	98.71 ± 0.052
nnU-Net	87.43 ± 0.102	92.42 ± 0.114	93.39 ± 0.132	93.03 ± 0.187	98.71 ± 0.064
TransUNet	86.06 ± 0.138	91.89 ± 0.123	**93.94 ± 0.117**	91.31 ± 0.094	98.62 ± 0.042
UCTransNet	85.81 ± 0.129	91.59 ± 0.183	93.48 ± 0.221	91.39 ± 0.237	98.46 ± 0.049
VM-UNet	85.26 ± 0.146	91.42 ± 0.160	92.18 ± 0.232	92.04 ± 0.178	98.75 ± 0.042
AC-MambaSeg	86.24 ± 0.121	91.85 ± 0.138	92.79 ± 0.205	92.57 ± 0.214	98.56 ± 0.034
H-vmunet	85.50 ± 0.151	91.58 ± 0.172	92.74 ± 0.120	91.97 ± 0.153	98.63 ± 0.046
UltraLight VM-UNet	83.47 ± 0.097	89.24 ± 0.139	89.37 ± 0.178	90.57 ± 0.118	96.84 ± 0.049
MFEVM-UNet	86.54 ± 0.108	92.25 ± 0.079	92.81 ± 0.091	92.52 ± 0.096	98.71 ± 0.038
DIG-MambaNet	**87.45 ± 0.155**	**93.02 ± 0.112**	93.36 ± 0.134	**93.46 ± 0.142**	**98.80 ± 0.062**

**Table 6:** Efficiency and accuracy comparison on the CVC-ClinicDB dataset at 256 × 256 resolution.

Model	mDice (%) ↑	Params (M) ↓	FLOPs (G) ↓	Inf. Time (ms/img) ↓	FPS (img/s) ↑	Peak VRAM (MB) ↓
U-Net	91.27 ± 0.108	31.038	54.738	2.949	339.073	240.6
Attention U-Net	90.58 ± 0.129	34.879	66.632	4.694	213.051	353.3
SE-Net	91.85 ± 0.093	7.821	13.754	4.927	202.948	237.6
nnU-Net	92.42 ± 0.114	33.472	115.786	5.243	190.720	190.4
TransUNet	91.89 ± 0.123	105.322	32.229	21.398	46.732	555.0
UCTransNet	91.59 ± 0.183	66.488	43.058	17.570	56.915	857.1
VM-UNet	91.42 ± 0.160	44.274	7.562	30.252	33.056	256.0
AC-MambaSeg	91.85 ± 0.138	7.994	6.273	22.177	45.091	646.1
H-vmunet	91.58 ± 0.172	8.973	0.742	95.612	10.459	347.1
UltraLight VM-UNet	89.24 ± 0.139	0.049	0.065	15.924	62.798	139.0
MFEVM-UNet	92.25 ± 0.079	61.747	12.665	35.709	28.004	590.8
DIG-MambaNet	93.02 ± 0.112	25.054	8.910	22.917	43.636	616.4

Note: ↑ indicates that higher values are preferable for mDice and FPS, whereas ↓ indicates that lower values are preferable for Params, FLOPs, inference time, and peak VRAM.

**Table 7:** Ablation experiments on the 2018DSB dataset.

Model	mIoU	mDice	mPrecision	mRecall	mAcc
*w*/*o* CSIM	86.57	92.48	92.67	93.23	97.83
*w*/*o* SIGM	86.77	92.58	92.58	92.84	97.83
*w*/*o* IDRFM	86.85	92.63	92.29	93.88	97.86
*w*/*o* CNN branch	86.15	92.16	92.42	92.97	97.74
*w*/*o* Mamba branch	86.66	92.43	92.64	93.08	97.84
Ours	**88.15**	**93.05**	**92.73**	**94.34**	**98.06**

**Table 8:** Ablation experiments on the JSUAH-Cerebellum dataset.

Model	mIoU	mDice	mPrecision	mRecall	mAcc
*w*/*o* CSIM	85.81	92.23	91.59	92.83	99.70
*w*/*o* SIGM	86.16	92.35	90.61	92.81	99.71
*w*/*o* IDRFM	86.52	92.66	93.09	92.63	99.72
*w*/*o* CNN branch	85.62	92.08	91.45	92.75	99.69
*w*/*o* Mamba branch	86.20	92.47	91.79	93.03	99.71
Ours	**87.93**	**93.13**	**93.78**	**93.48**	**99.74**

**Table 9:** Ablation experiments on the CVC-ClinicDB dataset.

Model	mIoU	mDice	mPrecision	mRecall	mAcc
*w*/*o* CSIM	84.91	91.43	91.62	91.96	98.33
*w*/*o* SIGM	85.03	91.41	92.14	91.61	98.13
*w*/*o* IDRFM	85.16	91.81	91.76	91.95	98.54
*w*/*o* CNN branch	84.80	91.29	91.37	91.28	98.44
*w*/*o* Mamba branch	85.62	91.62	91.62	93.08	98.51
Ours	**87.45**	**93.02**	**93.36**	**93.46**	**98.80**

**Table 10:** Ablation comparison of CSIM and representative attention mechanisms on the JSUAH-Cerebellum dataset.

Model	mIoU	mDice	mPrecision	mRecall	mAcc
*w*/*o* CSIM	85.81	92.23	91.59	92.83	99.70
CSIM → SE	86.75	92.80	92.88	93.11	99.73
CSIM → CBAM	86.68	92.78	92.94	92.96	99.73
CSIM → CoordAtt	87.30	92.83	93.14	93.76	99.73
Ours	87.93	93.13	93.78	93.48	99.74

**Table 11:** Effect of input image size on DIG-MambaNet performance and computational cost on the CVC-ClinicDB dataset.

Input Size	mIoU (%) ↑	mDice (%) ↑	mPrecision (%) ↑	mRecall (%) ↑	mAcc (%) ↑	FLOPs (G) ↓	FPS (img/s) ↑
224 × 224	86.78	92.19	92.92	92.46	98.78	6.82	43.81
256 × 256	87.45	93.02	93.36	93.46	98.80	8.91	43.64
320 × 320	87.64	93.00	94.29	92.68	98.80	13.92	41.75
384 × 384	87.75	93.01	94.02	93.11	98.80	20.04	41.45

Note: ↑ indicates that higher values are preferable for mIoU, mDice, mPrecision, mRecall, mAcc, and FPS, whereas ↓ indicates that lower values are preferable for FLOPs.

**Table 12:** Ablation study of different loss functions on the CVC-ClinicDB dataset.

Loss Function	mIoU	mDice	mPrecision	mRecall	mAcc
Dice loss	85.09	91.88	92.57	91.24	98.14
Dice+BCE loss	86.04	92.33	92.60	91.58	98.50
Focal loss	86.24	92.10	92.44	92.36	98.53
PPA loss	87.45	93.02	93.36	93.46	98.80

## Data Availability

The public datasets analyzed in this study are available from their original public repositories. The 2018DSB dataset is available at https://bbbc.broadinstitute.org/BBBC038 (accessed on 27 May 2026). The ISIC2018 dataset is available from the ISIC Challenge archive at https://challenge.isic-archive.com/data/ (accessed on 27 May 2026). The CVC-ClinicDB dataset is available at https://www.kaggle.com/datasets/balraj98/cvcclinicdb (accessed on 27 May 2026).

## References

[1] Litjens G., Kooi T., Bejnordi B.E., Setio A.A.A., Ciompi F., Ghafoorian M., Van Der Laak J.A., Van Ginneken B., Sánchez C.I. (2017). A Survey on Deep Learning in Medical Image Analysis. Med. Image Anal..

[2] Ronneberger O., Fischer P., Brox T., Navab N., Hornegger J., Wells W.M., Frangi A.F. (2015). U-Net: Convolutional networks for biomedical image segmentation. Medical Image Computing and Computer-Assisted Intervention—MICCAI 2015.

[3] Zhou Z., Siddiquee M.M.R., Tajbakhsh N., Liang J., Stoyanov D., Taylor Z., Carneiro G., Syeda-Mahmood T., Martel A., Maier-Hein L., Tavares J.M.R.S., Bradley A., Papa J.P., Belagiannis V. (2018). UNet++: A nested U-Net architecture for medical image segmentation. Deep Learning in Medical Image Analysis and Multimodal Learning for Clinical Decision Support.

[4] Oktay O., Schlemper J., Le Folgoc L., Lee M., Heinrich M., Misawa K., Mori K., McDonagh S., Hammerla N.Y., Kainz B. (2018). Attention U-Net: Learning where to look for the pancreas. arXiv.

[5] Luo W., Li Y., Urtasun R., Zemel R. (2016). Understanding the effective receptive field in deep convolutional neural networks. Adv. Neural Inf. Process. Syst..

[6] Xie X., Wu X., Pan L., Hua W., Shu X. (2026). CASA-Net: A context-aware and self-attention network for medical image segmentation. Biomed. Signal Process. Control.

[7] Shu X., Zhang A., Xu Z., Zhu F., Hua W. (2025). Adaptive encoding and comprehensive attention decoding network for medical image segmentation. Appl. Soft Comput..

[8] Shu X., Shi A., Guo X., Fan Y., Zhang X. (2026). Hierarchical attentive feature refinement network with cross-resolution detail preservation for medical image segmentation. Appl. Soft Comput..

[9] Dosovitskiy A., Beyer L., Kolesnikov A., Weissenborn D., Zhai X., Unterthiner T., Dehghani M., Minderer M., Heigold G., Gelly S. (2020). An image is worth 16 × 16 words: Transformers for image recognition at scale. arXiv.

[10] Vaswani A., Shazeer N., Parmar N., Uszkoreit J., Jones L., Gomez A.N., Kaiser L., Polosukhin I. (2017). Attention is all you need. Adv. Neural Inf. Process. Syst..

[11] Chen J., Lu Y., Yu Q., Luo X., Adeli E., Wang Y., Lu L., Yuille A.L., Zhou Y. (2021). TransUNet: Transformers make strong encoders for medical image segmentation. arXiv.

[12] Cao H., Wang Y., Chen J., Jiang D., Zhang X., Tian Q., Wang M., Karlinsky L., Michaeli T., Nishino K. (2023). Swin-Unet: Unet-like pure Transformer for medical image segmentation. Computer Vision—ECCV 2022 Workshops.

[13] Valanarasu J.M.J., Oza P., Hacihaliloglu I., Patel V.M., De Bruijne M., Cattin P.C., Cotin S., Padoy N., Speidel S., Zheng Y., Essert C. (2021). Medical Transformer: Gated axial-attention for medical image segmentation. Medical Image Computing and Computer Assisted Intervention—MICCAI 2021.

[14] Gu A., Dao T. Mamba: Linear-time sequence modeling with selective state spaces. Proceedings of the First Conference on Language Modeling.

[15] Ma J., Li F., Wang B. (2024). U-Mamba: Enhancing long-range dependency for biomedical image segmentation. arXiv.

[16] Yue Y., Li Z. (2024). MedMamba: Vision Mamba for medical image classification. arXiv.

[17] Xu J. (2024). HC-Mamba: Vision Mamba with hybrid convolutional techniques for medical image segmentation. arXiv.

[18] Milletari F., Navab N., Ahmadi S.A. (2016). V-Net: Fully convolutional neural networks for volumetric medical image segmentation. Proceedings of the 2016 Fourth International Conference on 3D Vision, Stanford, CA, USA, 25–28 October 2016.

[19] He K., Zhang X., Ren S., Sun J. Deep residual learning for image recognition. Proceedings of the IEEE Conference on Computer Vision and Pattern Recognition.

[20] Huang G., Liu Z., van der Maaten L., Weinberger K.Q. Densely connected convolutional networks. Proceedings of the IEEE Conference on Computer Vision and Pattern Recognition.

[21] Howard A.G., Zhu M., Chen B., Kalenichenko D., Wang W., Weyand T., Andreetto M., Adam H. (2017). MobileNets: Efficient convolutional neural networks for mobile vision applications. arXiv.

[22] Sandler M., Howard A., Zhu M., Zhmoginov A., Chen L.C. MobileNetV2: Inverted residuals and linear bottlenecks. Proceedings of the IEEE Conference on Computer Vision and Pattern Recognition.

[23] Hu J., Shen L., Sun G. Squeeze-and-Excitation Networks. Proceedings of the IEEE Conference on Computer Vision and Pattern Recognition.

[24] Woo S., Park J., Lee J.Y., Kweon I.S. CBAM: Convolutional Block Attention Module. Proceedings of the European Conference on Computer Vision.

[25] Roy A.G., Navab N., Wachinger C., Frangi A.F., Schnabel J.A., Davatzikos C., Alberola-López C., Fichtinger G. (2018). Concurrent Spatial and Channel Squeeze & Excitation in Fully Convolutional Networks. Medical Image Computing and Computer Assisted Intervention—MICCAI 2018.

[26] Misra D., Nalamada T., Arasanipalai A.U., Hou Q. Rotate to Attend: Convolutional Triplet Attention Module. Proceedings of the IEEE/CVF Winter Conference on Applications of Computer Vision.

[27] Hou Q., Zhou D., Feng J. Coordinate Attention for Efficient Mobile Network Design. Proceedings of the IEEE/CVF Conference on Computer Vision and Pattern Recognition.

[28] Wang H., Cao P., Wang J., Zaiane O.R. (2022). UCTransNet: Rethinking the skip connections in U-Net from a channel-wise perspective with Transformer. Proc. AAAI Conf. Artif. Intell..

[29] Liu Z., Lin Y., Cao Y., Hu H., Wei Y., Zhang Z., Lin S., Guo B. Swin Transformer: Hierarchical vision Transformer using shifted windows. Proceedings of the IEEE/CVF International Conference on Computer Vision.

[30] Wang W., Xie E., Li X., Fan D.P., Song K., Liang D., Lu T., Luo P., Shao L. Pyramid Vision Transformer: A versatile backbone for dense prediction without convolutions. Proceedings of the IEEE/CVF International Conference on Computer Vision.

[31] Zhu L., Liao B., Zhang Q., Wang X., Liu W., Wang X. (2024). Vision Mamba: Efficient visual representation learning with bidirectional state space model. Proc. Mach. Learn. Res..

[32] Liu Y., Tian Y., Zhao Y., Yu H., Xie L., Wang Y., Ye Q., Jiao J., Liu Y. (2024). VMamba: Visual state space model. Adv. Neural Inf. Process. Syst..

[33] Ruan J., Li J., Xiang S. (2025). VM-UNet: Vision Mamba UNet for medical image segmentation. ACM Trans. Multimed. Comput. Commun. Appl..

[34] Chen C., Yu L., Min S., Wang S. MSVM-UNet: Multi-scale Vision Mamba UNet for medical image segmentation. Proceedings of the 2024 IEEE International Conference on Bioinformatics and Biomedicine.

[35] Wu R., Liu Y., Liang P., Chang Q. (2025). H-vmunet: High-order Vision Mamba UNet for medical image segmentation. Neurocomputing.

[36] Wu R., Liu Y., Ning G., Liang P., Chang Q. (2025). UltraLight VM-UNet: Parallel Vision Mamba significantly reduces parameters for skin lesion segmentation. Patterns.

[37] Liu J., Yang H., Zhou H.-Y., Xi Y., Yu L., Li C., Liang Y., Shi G., Yu Y., Zhang S., Linguraru M.G., Dou Q., Feragen A., Giannarou S., Glocker B., Lekadir K., Schnabel J.A. (2024). Swin-UMamba: Mamba-based UNet with ImageNet-based pretraining. Medical Image Computing and Computer Assisted Intervention—MICCAI 2024.

[38] Wu X., Huang S., Shu X., Hu C., Wu X.J. (2024). MPFC-Net: A multi-perspective feature compensation network for medical image segmentation. Expert Syst. Appl..

[39] Wei J., Wang S., Huang Q. (2020). F^3^Net: Fusion, feedback and focus for salient object detection. Proc. AAAI Conf. Artif. Intell..

[40] Qin X., Zhang Z., Huang C., Gao C., Dehghan M., Jagersand M. BASNet: Boundary-aware salient object detection. Proceedings of the IEEE/CVF Conference on Computer Vision and Pattern Recognition.

[41] Wei J., Zhu G., Fan Z., Liu J., Rong Y., Mo J., Li W., Chen X. (2022). Genetic U-Net: Automatically designed deep networks for retinal vessel segmentation using a genetic algorithm. IEEE Trans. Med. Imaging.

[42] Caicedo J.C., Goodman A., Karhohs K.W., Cimini B.A., Ackerman J., Haghighi M., Heng C.K., Becker T., Doan M., McQuin C. (2019). Nucleus segmentation across imaging experiments: The 2018 Data Science Bowl. Nat. Methods.

[43] Tschandl P., Rosendahl C., Kittler H. (2018). The HAM10000 dataset, a large collection of multi-source dermatoscopic images of common pigmented skin lesions. Sci. Data.

[44] Shu X., Gu Y., Zhang X., Hu C., Cheng K. (2022). FCRB U-Net: A novel fully connected residual block U-Net for fetal cerebellum ultrasound image segmentation. Comput. Biol. Med..

[45] Bernal J., Sánchez F.J., Fernández-Esparrach G., Gil D., Rodríguez C., Vilariño F. (2015). WM-DOVA maps for accurate polyp highlighting in colonoscopy: Validation vs. saliency maps from physicians. Comput. Med. Imaging Graph..

[46] Nguyen V.T., Pham V.T., Tran T.T., Huang Y.P., Wang W.J., Le H.G., Hoang A.Q. (2024). AC-MambaSeg: An adaptive convolution and Mamba-based architecture for enhanced skin lesion segmentation. Computational Intelligence Methods for Green Technology and Sustainable Development.

[47] Guo F., Zhang S., Sun Z., Zhang L., Guo J., Lu X., Chen X. (2026). MFEVM-UNet: Multi-scale feature fusion and enhancement Vision Mamba UNet for medical image segmentation. Biomed. Signal Process. Control.

[48] Isensee F., Jaeger P.F., Kohl S.A.A., Petersen J., Maier-Hein K.H. (2021). nnU-Net: A self-configuring method for deep learning-based biomedical image segmentation. Nat. Methods.

